# PH CARE COVID survey: an international patient survey on the care for pulmonary hypertension patients during the early phase of the COVID-19 pandemic

**DOI:** 10.1186/s13023-021-01752-1

**Published:** 2021-05-01

**Authors:** Laurent Godinas, Keerthana Iyer, Gergely Meszaros, Rozenn Quarck, Pilar Escribano-Subias, Anton Vonk Noordegraaf, Pavel Jansa, Michele D’Alto, Milan Luknar, Senka Milutinov Ilic, Catharina Belge, Olivier Sitbon, Abílio Reis, Stephan Rosenkranz, Joanna Pepke-Zaba, Marc Humbert, Marion Delcroix

**Affiliations:** 1 Department of Respiratory Diseases, University Hospitals Leuven, Leuven, Belgium; 2 Laboratory of Respiratory Diseases and Thoracic Surgery (BREATHE), Department of Chronic Diseases and Metabolism (CHROMETA), KU Leuven - University of Leuven, Leuven, Belgium; 3 Faculté de Médecine, Université Paris-Saclay, Le Kremlin Bicêtre, France; 4 Assistance Publique Hôpitaux de Paris, Service de Pneumologie, Centre de Référence de L’Hypertension Pulmonaire, ERN-LUNG, Hôpital Bicêtre, Le Kremlin Bicêtre, France; 5 Inserm UMR_S 999, Hôpital Marie Lannelongue, Le Plessis Robinson, France; 6 European Pulmonary Hypertension Association, Vienna, Austria; 7 Cardiology Department and Spanish Cardiovascular Research Network (CIBER-CV), Hospital Universitario, 12 de Octubre, Madrid, Spain; 8 Departement of Pulmonary Medicine, Vrije Universiteit Amsterdam, Amsterdam UMC, De Boelelaan 1117, Amsterdam, Netherlands; 9 Department of Medicine - Department of Cardiovascular Medicine, First Faculty of Medicine, Charles University and General University Hospital, Prague, Czech Republic; 10 Department of Cardiology, Monaldi Hospital, Naples, Italy; 11 National Cardiovascular Institute, Comenius University School of Medicine, Pod Krasnou Horkou 1, Bratislava, Slovakia; 12 Institute for Pulmonary Diseases of Vojvodina, Sremska Kamenica, Serbia; 13 Medicine Department, Pulmonary Vascular Diseases Unit, Centro Hospitalar Universitário Do Porto, Porto, Portugal; 14 Department III of Internal Medicine and, Cologne Cardiovascular Research Center (CCRC), Cologne University Heart Center, Cologne, Germany; 15 Pulmonary Vascular Diseases Unit, Royal Papworth Hospital, Cambridge, UK

**Keywords:** Pulmonary hypertension, COVID-19, Patient survey, Pulmonary arterial hypertension, Chronic thromboembolic pulmonary hypertension

## Abstract

**Background:**

During the COVID-19 pandemic, most of the health care systems suspended their non-urgent activities. This included the cancellation of consultations for patients with rare diseases, such as severe pulmonary hypertension (PH), resulting in potential medication shortage and loss of follow-up. Thus, the aim of the study was to evaluate PH patient health status evolution, access to health care and mental health experience during the early phase of the pandemic.

**Methods:**

We conducted an online patient survey, available in 16 languages, between 22/05/2020 and 28/06/2020. The survey included questions corresponding to demographic, COVID-19 and PH related information.

**Results:**

1073 patients (or relatives, 27%) from 52 countries all over the world participated in the survey. Seventy-seven percent (77%) of responders reported a diagnosis of pulmonary arterial hypertension and 15% of chronic thromboembolic PH. The COVID-19 related events were few: only 1% of all responders reported a diagnosis of COVID-19. However, 8% of patients reported health deterioration possibly related to PH, and 4% hospitalization for PH. Besides, 11% of the patients reported difficulties to access their PH expert centre, and 3% interruption of treatment due to shortage of medication. Anxiety or depression was reported by 67% of the participants.

**Conclusion:**

Although COVID-19 incidence in PH patients was low, PH related problems occurred frequently as the pandemic progressed, including difficulties to have access to specialized care. The importance of primary health care was emphasized. Further studies are needed to evaluate the long-term consequences of COVID-related PH care disruption.

**Supplementary Information:**

The online version contains supplementary material available at 10.1186/s13023-021-01752-1.

## Introduction

SARS-CoV-2 (severe acute respiratory syndrome coronavirus 2) emerged in China at the end of 2019 causing coronavirus disease 2019 (COVID-19). The outbreak rapidly spread around the world and on the 11th of March 2020, the World Health Organization declared that COVID-19 could be characterized as a pandemic. COVID-19 reached Europe in February 2020 resulting in a strict lockdown in most European countries between March and June 2020. During these three months, the healthcare systems encountered difficulties to ensure non-urgent care due to COVID-19 patient overload. A substantial part of outpatient consultations for patients with chronic or rare diseases were cancelled, with potential repercussions on their health status. In the meantime, these patients were considered to be at risk for severe forms of COVID-19 due to their underlying condition, especially in the case of patients with cardio-pulmonary diseases.

Pulmonary arterial hypertension (PAH) and other severe forms of pulmonary hypertension (PH) such as chronic thromboembolic pulmonary hypertension (CTEPH), are rare diseases characterized by an elevated mean pulmonary arterial pressure above 20 mmHg, normal pulmonary artery wedge pressure, and elevated pulmonary vascular resistance above 3 Wood Units, due to progressive precapillary pulmonary artery remodelling [1, 2]. They require highly specialized care in expert centers and treatment with PAH oral drugs, sometimes combined with inhaled, continuous intravenous or subcutaneous agents [3]. CTEPH patients could also benefit from surgical or interventional treatments. These complex treatments require frequent monitoring by specialized physicians and nurses. Furthermore, most of these drugs are expensive and not easily accessible due to health system restrictions.

PH patients were subjected to the same rules as other patients during the COVID-19 pandemic, with no prioritized care despite their rare and severe condition. Most of the PH centres, mainly run by cardiologists or respiratory physicians, suddenly stopped their activities considered non-urgent in order to focus on COVID-19 patient care, generally in response to directives from the local/national authorities. The difficulties to access specialized health care and specific drugs suggest that the interruption of care could have major repercussions on the health status of PH patients [4]. To get a rapid picture of the situation and problems encountered, a consortium composed of PH patient associations and scientific societies was created. The European Pulmonary Hypertension Association (PHA Europe), the European Reference Network for rare lung diseases (ERN-LUNG), the European Respiratory Society (ERS) Assembly 13 on Pulmonary Vascular Diseases, the ERS Clinical Research Collaboration PHAROS, the European Lung Foundation (ELF) and the European Society of Cardiology (ESC) Working Group on Pulmonary Circulation & Right Ventricular Function decided to join forces to launch the PH-CARE-COVID survey, an international patient survey available in 16 languages, designed to collect information on PH patients’ lived experience and to understand how PH care was provided during the pandemic-related lockdown.

## Methods

### Survey design and dissemination

An online survey of 34 questions was created regarding: (1) the demographics and disease related information; (2) the COVID experience of PH patients; and (3) the PH disease management during the COVID-19 pandemic. The original English version of the questionnaire is available in the Additional file 1.

The survey was translated from English into 15 languages (Bulgarian, Czech, Dutch, French, German, Hebrew, Italian, Latvian, Lithuanian, Portuguese, Serbian, Slovak, Slovenian, Spanish, Ukrainian). The translations were made by experienced native speakers from national patient associations and validated by a local PH specialist physician. The questionnaire was created using the “Survey Monkey” tool and made accessible on the PHA Europe website (https://www.phaeurope.org/get-involved/advocacy-policy-work/covid-19-questionnaire/) and on the ERN-LUNG website (https://ern-lung.eu/portfolio-items/phcare-covid-survey/). The survey was disseminated by the European PH umbrella association, national PH patient associations and by PH centres through their websites, social networks and other suitable media. In addition, the ERS Assembly 13 on Pulmonary Vascular Diseases and the ESC Working Group on Pulmonary Circulation & Right Ventricular Function specifically informed their affiliated PH physicians. An information folder concerning the survey was created that could be distributed to patients by the PH centres.

The survey was anonymous, however patients had the possibility to give an email address to receive further information, feedback of the study or other studies concerning the COVID-19 pandemic. There were no exclusion criteria concerning the type of PH (group 1 to 5). For some questions, more than one answer was possible.

### Income level determination

Income level per country was based on the Gross National Income (GNI) per capita calculated using the World Bank Atlas Method and the data available on the website of Word Bank, data provided in US dollar per year [5]. Low-income economies are defined as those with a GNI per capita of $1,035 or less in 2019; middle-income economies are those with a GNI per capita between $1,036 and $12,535; high-income economies are those with a GNI per capita of $12,536 or more).

### Data collection and analysis

The survey was accessible between 22/05/2020 and 28/06/2020. The results were extracted from the “Survey Monkey” tool on an Excel sheet and were analysed using GraphPad Prism (v. 8). Google Translate was used to analyse open answers from participants when necessary.

## Results

### Patient characteristics

**Fig. 1:**
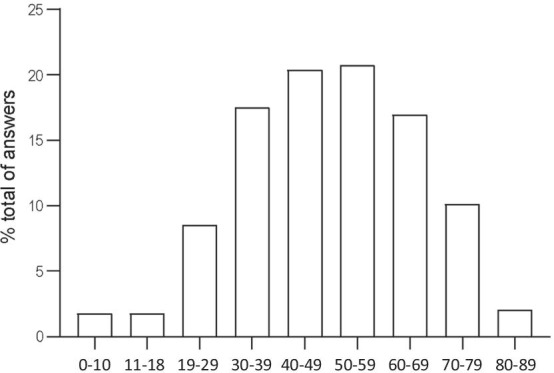
Age distribution of the patients

A total of 1073 individuals answered the survey. 73% were patients and 27% relatives of patients. They came from 52 countries (Table 1), with the largest number of participants in Belgium (n = 144, 13.4%), France (n = 125, 11.6%), the Netherlands (n = 116, 10.8%), Chile (n = 90, 8.4%), and Spain (n = 78, 7.3%). Demographic data are presented in Table 2. Most of the cohort were adult (96%) and 4% were paediatric patients (Fig. 1). The majority reported to have idiopathic PAH (39%), followed by congenital heart disease associated PAH (19%) and by CTEPH (15%). In total, 92% of the patients reported a diagnostic of PAH or CTEPH. Some patients could not provide an accurate diagnosis (6%) and 1% gave answers other than PH group 1 and 4. Most of the patients (87%) received oral therapy and 21% received parenteral therapy. The questionnaire did not allow to differentiate monotherapy from dual or triple combination therapy. Eleven percent of the patients denied receiving specific therapies, mostly corresponding to operated CTEPH patients, patients treated with calcium channel blockers, patients with ongoing diagnosis, and patients with PH types other than group 1 or 4. The median follow up time in PH specialized centres was 4.5 years (IQ 2.0–10.0).

**Table 1:** Distribution of the participants per country

Name of country	Total answers per country	% of the total answers of the survey
Belgium	144	13.4
France	125	11.6
The Netherlands	116	10.8
Chile	90	8.4
Spain	78	7.3
Ukraine	74	7.0
Portugal	66	6.2
Italy	47	4.4
Austria	37	3.4
Germany	29	2.7
Serbia	24	2.2
Latvia	23	2.1
Slovakia	21	2.0
Slovenia	18	1.7
Canada	17	1.6
India	17	1.6
United Kingdom	15	1.4
Brazil	13	1.2
Colombia	12	1.1
Czechia	12	1.1
USA	9	0.8
Bosnia	9	0.8
Croatia	9	0.8
Lithuania	8	0.7
Argentina	7	0.7
Peru	7	0.7
Uruguay	6	0.6
Australia	4	0.4
Bulgaria	4	0.4
Finland	3	0.3
Dominican Republic	3	0.3
Norway	2	0.2
Poland	2	0.2
Sweden	2	0.2
Honduras	2	0.2
Greece	2	0.2
New Zealand	1	< 0.1
Mexico	1	< 0.1
Montenegro	1	< 0.1
El Salvador	1	< 0.1
Costa Rica	1	< 0.1
Ecuador	1	< 0.1
Venezuela	1	< 0.1
Switzerland	1	< 0.1
Algeria	1	< 0.1
Morocco	1	< 0.1
Malaysia	1	< 0.1
South Africa	1	< 0.1
Russia	1	< 0.1
Hungary	1	< 0.1
Lebanon	1	< 0.1
Tajikistan	1	< 0.1

**Table 2:** Demographics, PH etiology and PH specific therapies. *PAH* pulmonary arterial hypertension, *PH* pulmonary hypertension

	N = 1073	%
*Gender*		
Female	839	78.2
Male	228	21.2
No answer	6	0.6
*PH etiology*		
Idiopathic PAH	416	38.8
Heritable/genetic PAH	80	7.5
Drug related PAH	15	1.4
Liver disease related PAH	16	1.5
Connective tissue disease related PAH	93	8.7
Congenital heart malformation related PAH	208	19.4
Chronic thromboembolic PH	161	15.0
Other	13	1.2
I do not know/I am not sure/Do not want to answer	71	6.6
*Treatments*		
Oral	934	87.0
Intravenous	76	7.1
Subcutaneous	77	7.2
Inhaled	72	6.7
Study medication	18	1.7
No specific PH treatment reported	115	10.7

### COVID-19 experience of PH patients

**Fig. 2:**
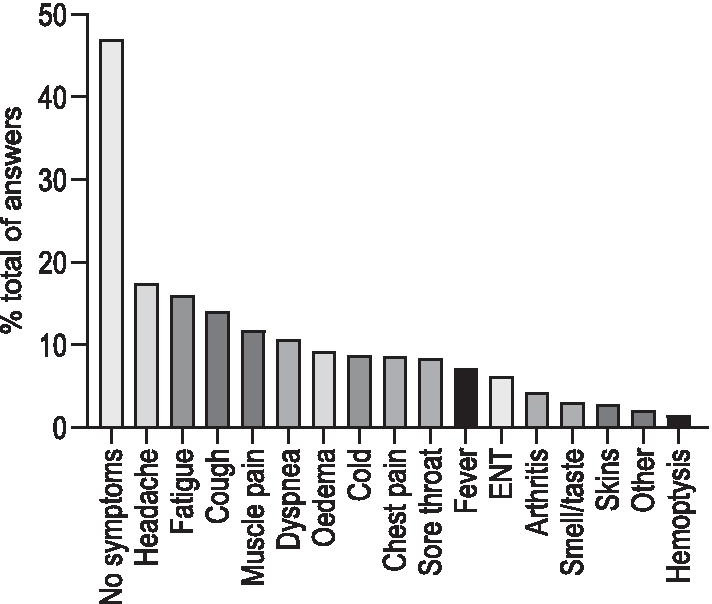
Patients reported symptoms during the period of the lockdown. ENT, ear, nose and throat symptoms

**Fig. 3:**
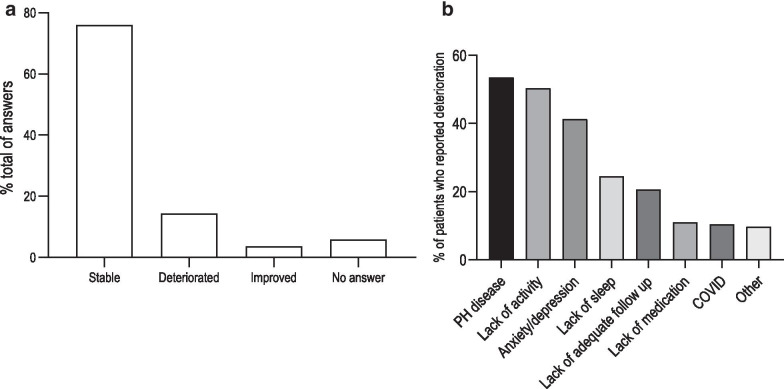
**a** Evolution of self-reported health status during the lockdown. **b** Self-reported causes of deterioration for patients who reported a deterioration of their health status. *PH* pulmonary hypertension

This part of the questionnaire aimed to understand whether PH patients experienced any COVID-19 related events (appearance of symptoms, testing, hospitalization) and their primary source of contact during this experience. During the early phase of the COVID-19 pandemic, 47% of the patients had no new symptom or aggravation of previously known symptoms (Fig. 2). The majority of the symptoms reported by patients were unspecific but compatible with viral diseases or PH (Fig. 2). Three percent of the patients reported a loss of smell or taste. 815 (76%) patients judged their physical condition as stable, 155 (14%) felt a deterioration and 40 (4%) an improvement, while 63 (6%) did not answer the question (Fig. 3a). In the 155 patients who experienced a deterioration, 54% attributed it to PH (8% of total participants), 50% to a lack of activity due to lockdown (7% of total), 41% to anxiety and depression (6% of total), 11% (2% of total) to the lack of medications and only 10% directly to COVID (1% of total) (Fig. 3b).

**Fig. 4:**
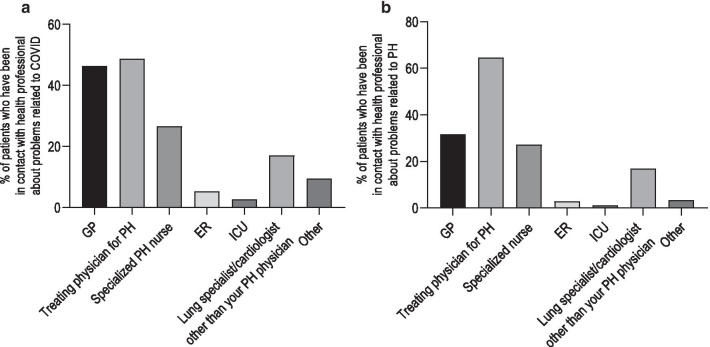
**a** Patients self-reported contacts with health professionals concerning COVID-related issues. **b** Patients self-reported contacts with health professionals concerning PH-related issues. *ER* emergency room, *GP* general practitioner, *ICU* intensive care unit, *PH* pulmonary hypertension

Most of the patients (756, 70%) reported not to have been in contact with health professionals for problems or questions related to COVID, while 263 patients (25%) reportedly contacted health professionals for COVID related reasons. 54 patients (5%) did not answer this question. Out of the 263 patients who reported contact, 122 (46%) reported a contact with their general practitioner (GP), 128 (49%) with their treating PH physician, 70 (27%) with their specialized PH nurses, 45 (17%) with a cardiologist or a pneumologist other than their PH physician, 21 (8%) with emergency/intensive care unit (ICU) physicians and 25 (10%) with other health professionals (mainly specialists or nurses outside of the specialist centres) (Fig. 4a).

918 (86%) patients were not tested for COVID-19, while 104 (10%) were. Out of the 10% who were tested, 90% of the tests were negative and only 9% positive, corresponding to 9 patients who tested positive for COVID-19. 13 patients (1%) reported hospitalization for COVID-19, of which with 4 patients were hospitalized in their own PH centre and 7 in centres other than their respective PH centres. Only 2 patients reported hospitalization in intensive care units. The median length of stay in hospital was 6 days (min: 2, max: 43).

### Impact of COVID-19 on PH care

This part of the questionnaire aimed to understand the possible impact of COVID-19 on PH care continuity and health status evolution. 475 (44%) participants were in contact with health professionals concerning questions or problems related to PH, 535 (50%) were not and 63 (6%) did not answer. From the 44% of patients who reported contact, 150 (32%) reported a contact with their GP, 307 (65%) with their treating PH physician, 129 (27%) with their specialized nurse team, 80 (17%) with a cardiologist or a pneumologist other than their PH physician, 18 (4%) with emergency/ICU physicians and 16 (3%) with other health professionals (mainly specialists or nurses outside of the specialist centres) (Fig. 4b). Among patients who did not report any contact, no consultations were planned for 317 of them (59%), 41 (8%) were afraid to disturb their physicians, 111 (21%) were afraid to go to a public place/hospital, 73 (14%) did not consider their problem to be important and 57 (11%) for other reasons. During the early phase of the COVID-19 pandemic, 959 (89%) patients reported no PH-related hospitalization, 43 (4%) were hospitalized due to their PH condition, and 71 (6%) did not answer. Among the 43 who were hospitalized, 30 were hospitalized in their own PH centre (emergency room = 5, general ward = 21, ICU = 3) and 11 reported to have been hospitalized in another centre (emergency room = 4, general ward = 4, ICU = 3). The median length of hospitalization was 7 days (min: 1, max: 83). Survey participants were also asked if they had trouble to reach health professionals or to have access to healthcare services during the early phase of the COVID-19 pandemic. 117 (11%) reported difficulties to join their PH treating team, 101 (9%) to join health professionals other than their PH team, 175 (16%) to obtain adequate information related to the repercussion of COVID-19 on PH and 172 (16%) to receive their PH specific medications. PH patients were also asked whether they experienced cancellation of their appointments/medical check-ups and how the rescheduling was managed. 448 (42%) experienced no cancellation while 530 (49%) did. Among those who did, 342 (32%) reported cancellation of consultation with their PH team and 377 (35%) reported cancellation of exams related to PH. For those who experienced cancellation, 338 (64%) reported to have had their appointments cancelled by their PH centre, 87 (16%) decided to cancel by themselves and 108 (20%) decided in concertation with their PH physician; 138 patients (13% of the total of answering patients) reported not having received any new appointment or other instructions concerning rescheduling.

130 (12%) patients reported to have reached their PH team by face-to-face contact, 455 (42%) by phone call, 83 (8%) by video call, 280 (26%) via email and 65 (6%) only via post.

Specific questions concerning the continuity of treatments were also asked: 870 (81%) patients reported no change in their treatment, 27 (3%) reported a treatment interruption due to shortage of medication, 6 (1%) stopped for reasons other than shortage and 41 (4%) reported a modification of their treatment. Among the patients who stopped their treatment due to shortage, the duration was less than one week for 6 (22%), between 1 and 4 weeks for 10 (37%), and more than 4 weeks for 11 (41%) patients. 7 patients reported stopping oral treatment and 2 stopped intravenous treatment. 4 patients reported the information to their PH centre and 5 to their GP. Out of the 27 patients who reported interruption of medication due to shortage, 37% were patients from high-income countries and 63% from middle-income countries.

**Fig. 5:**
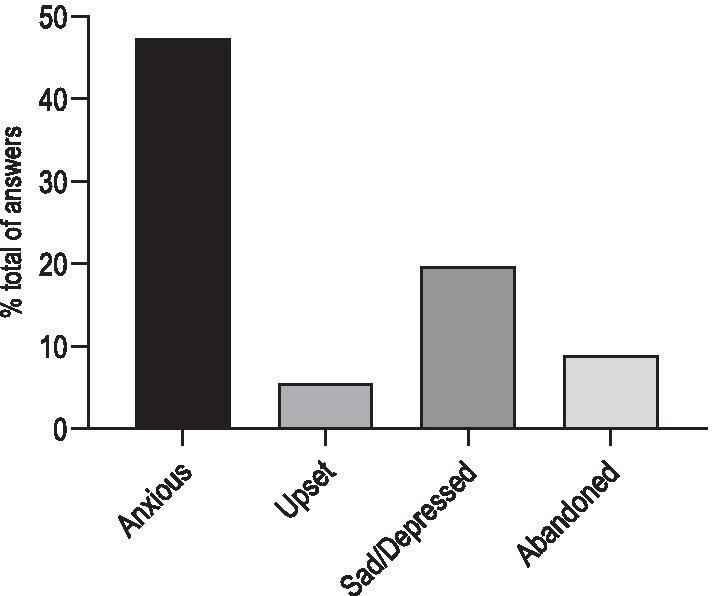
Self-reported mental status of patients during the lockdown

Concerning how they felt regarding their health status as PH patients during the pandemic, 508 (47%) patients experienced anxiety, 59 (5%) were upset, 211 (20%) reported sadness or depression and 95 (9%) felt abandoned (Fig. 5). Regarding the medical care that PH patients received during the early phase of the COVID-19 pandemic, 556 (52%) were satisfied or very satisfied, 200 (19%) were neutral and 59 (5%) were unsatisfied or very unsatisfied.

606 (56%) patients reported to have searched for specific information concerning the repercussions of COVID-19 on PH health status. For these patients, the main sources of information were the internet (n = 433, 71%), patient associations (n = 208, 34%), their PH treating team (n = 178, 29%), GP (n = 104, 17%) and other health professionals (n = 53, 9%).

Finally, 55% of the participants considered their PH centres to be well prepared to face the pandemic and 73% thought that PH patient associations could have a crucial role to play in such events in the future.

## Discussion

In this patient survey representing the experience of 1073 PH patients and their caregivers, we showed that (1) about 1% of the PH patients reported testing positive for COVID-19, while reporting of health status deterioration was tenfold higher and mainly related to PH, deconditioning and mood disorders; (2) reported hospitalizations were 4 times more frequent for PH than for COVID-19; (3) 50% of the patients stayed in contact remotely with their PH team, mainly via phone calls and e-mails; (4) continuity of care was not possible for a significant part of the patients regarding contacts with their PH team, absence of scheduled follow-up and shortage of medication; (5) primary health care system was frequently consulted regarding COVID-19 related problems; (6) finally, the internet and patient associations were the most frequently consulted sources by PH patients for information related to COVID-19.

Since the early days of the pandemic, cardiovascular and respiratory conditions have been associated with poor outcome in COVID-19 [6]. Moreover, the rapidly recognized nature of pulmonary vasculopathy [7] and high rate of venous thromboembolic events in COVID-19 patients [8] pointed towards increased risks of severity in PH patients [9]. In our series, less than 10 patients (1%) were diagnosed with COVID-19. A previous study by Lee et al. reported a lower incidence, with a COVID-19 rate of 2.1 per 1000 PH patients in the US, similar to the incidence in US global population, but with a high rate of mortality [10]. Belge et al. also reported a high mortality rate of 19% in PH patients with COVID-19 from a survey of PH centres [11]. In the current survey, incidence was higher but severity seemed lower. The difference in incidence could be explained by temporal and demographic considerations with higher incidence rate of COVID-19 infection in Europe than in the US in the early phase of the pandemic [12]. The lower severity of COVID-19 could be due to under-representation of severe COVID-19 cases, due to death or insufficient recovery at the time of the survey or older age. However, the incidence of 1% seems plausible. Indeed, in regions with high COVID-19 incidence, serological studies in the early pandemic phase have shown seroprevalence up to 23% in Lombardy [13], 7.6% in Korea [14], and 6.2% in Philadelphia, USA [15]. It is probable that most of the patients with cardio-pulmonary comorbidities have been very cautious regarding SARS-CoV-2 infection, strictly following social distancing and isolation recommendations because of awareness of risk of developing severe disease [16]. Moreover, 3% of the patients reported loss of smell, which could be related to less symptomatic SARS-CoV-2 infection [17]. Further serological studies and large-scale health systems data analysis are required to confirm the exact incidence of COVID-19 in PH populations. Concerning COVID-19-related hospitalization, we found a median duration of stay in hospital of 6 days, which seems relatively similar to a recent PH centre survey which reported 3 days [11]. This duration is compatible with a majority of moderate COVID-19 cases in PH patients but could hide quite large disparities, mainly for more severe patients requiring ICU hospitalizations.

The second major concern for PH patients was the reorganization of care during the lockdown which had considerable impact on other diseases [18], and especially in cardiovascular disease [19], with low access to healthcare system, prioritization of access to specific patients, lack of material or shortage of medications [20]. Worse outcomes due to delay in care has also been shown for acute cardiovascular problems [21]. However, there were some disparities between countries depending on country income [22, 24]. Our current survey provided a unique opportunity to assess this problem in PH patients. We found that a low but significant number of PH patients encountered difficulties to have access to the healthcare system during the lockdown, with difficulties to reach PH specialized physicians or to continue therapy due to shortage. Interestingly, two thirds of the patients who stopped their medication were patients from middle-income countries as opposed to one third from high-income countries, spreading light on healthcare system disparities during the lockdown [25]. In addition, some patients’ appointments were cancelled without new appointments or rescheduling instructions. This lack of continuity of care during the pandemic should be a specific issue to address in PH centers and specific policies should be implemented in prevision of new episodes of the pandemic [26].

An important proportion of the patients were able to maintain contact with their PH specialized physicians during the early phase of the pandemic, mainly via phone call and e-mail, allowing some health status monitoring. Other medical disciplines have shown different ways to stay in touch with patients to be successful [27, 28]. These solutions should be considered and telemedicine plans prepared for the future [20]. Interestingly, concerning COVID-19 related problems, the PH patients turned to their GP as frequently as to their PH physicians, reinforcing the role of the primary care system during the pandemic [29]. Therefore, effective communication between PH expert centres and primary health care system should occur regarding pandemic-related specific recommendations for patients with rare diseases. Moreover, patients reported the internet and PH patient associations as important sources of information. These specific means of communication should be recognized and largely used in case of a new pandemic.

Fourteen percent of the patients noted a deterioration of their health status during the early phase of the pandemic. Although probably multifactorial, this deterioration may be related to progression of the underlying pulmonary vascular disease due to a potential delay in adequate management [21]. The strategy of early identification of these patients in the resumption of activity and in case of further pandemics, should be implemented [30]. The role of deconditioning and mood disorders due to confinement should require attention. Rehabilitation program and remote psychological support should be proposed to patients who need it.

Although COVID-19 occupied centre stage during the early phase of the pandemic, the PH patients seem to have been hit 4 times more by PH related issues than by COVID-19. It is, to our knowledge, the first broad evaluation of problems encountered by PH patients and the first time that we can quantify and compare the proportion of PH related problems with COVID-19 related problems. Our data clearly show the importance of maintaining a continuity of care for PH patients and they clearly suggest that specific recommendations should be made to allow maintenance of good quality care in the specific context of a pandemic and lockdown conditions.

Our approach has some limitations. Firstly, it is a patient survey and no direct control exists on the data provided by patients, especially no medical control concerning the reporting of the clinical deterioration by the patients. However, the large number of answers is quite reassuring, ironing out the potential mistakes or inaccuracies in patients’ answers. Secondly, although we received a high number of answers, this survey was conducted around the world, resulting in quite a low number of answers from some countries, precluding in depth analysis and comparisons per country. However, taking into account the disparity in health systems, incomes and the timing of the pandemic, reporting on the global experience of the PH patients seems relevant and gives a unique overview of the worldwide impact of the pandemic on the continuity of care in PH. Finally, patients experiencing fatal or very severe outcomes after SARS-CoV-2 infection or PH clinical deterioration will not participate in this kind of survey. This precludes the collection of data in this specific subgroup highly impacted by the pandemic, constituting a bias that has to be taken into account.

In conclusion, PH-related problems were encountered more frequently than COVID-19-related problems during the early phase of the pandemic. Further studies are needed to evaluate the long-term consequences of COVID-19-related PH care disruption. Information on the impact of COVID-19 on various rare lung conditions must be made available to patients and caregivers, while remaining vigilant to prevent the spread of misinformation. The importance of primary healthcare even in the case of specialized rare conditions should not be undermined. This data could be of interest for further planning of strategies and organization of PH centres to ensure continuity of care and adequate communications with patients, caregivers, and health care providers, including primary care physicians.

## Supplementary Information

**Additional file 1.** English questionnaire of the survey.

**Additional File 2.** Complete results of the answers to the questionnaire.

## Data Availability

All the results of the survey are available in supplementary materials (Additional file 2).
