# Supplementary material for: PH CARE COVID survey: an international patient survey on the care for pulmonary hypertension patients during the early phase of the COVID-19 pandemic
Source: Orphanet J Rare Dis. 2021 May 1;16:196. doi: 10.1186/s13023-021-01752-1 (PMC8087873; doi:10.1186/s13023-021-01752-1)
Supplement: Supplementary file 1 — Additional file 1. English questionnaire of the survey. [file 13023_2021_1752_MOESM1_ESM.pptx]

## Slide 1
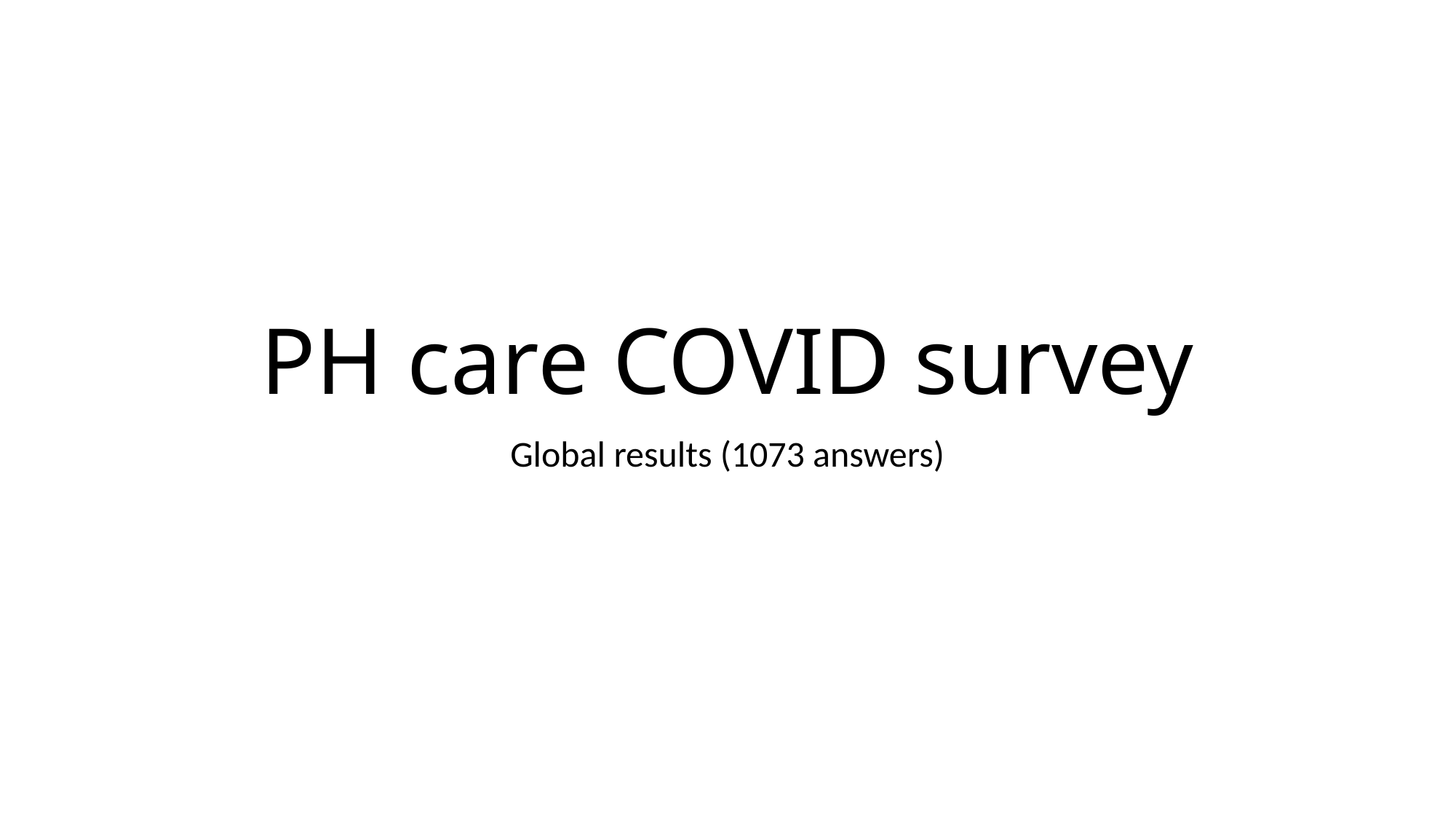

# PH care COVID survey
Global results (1073 answers)

## Slide 2
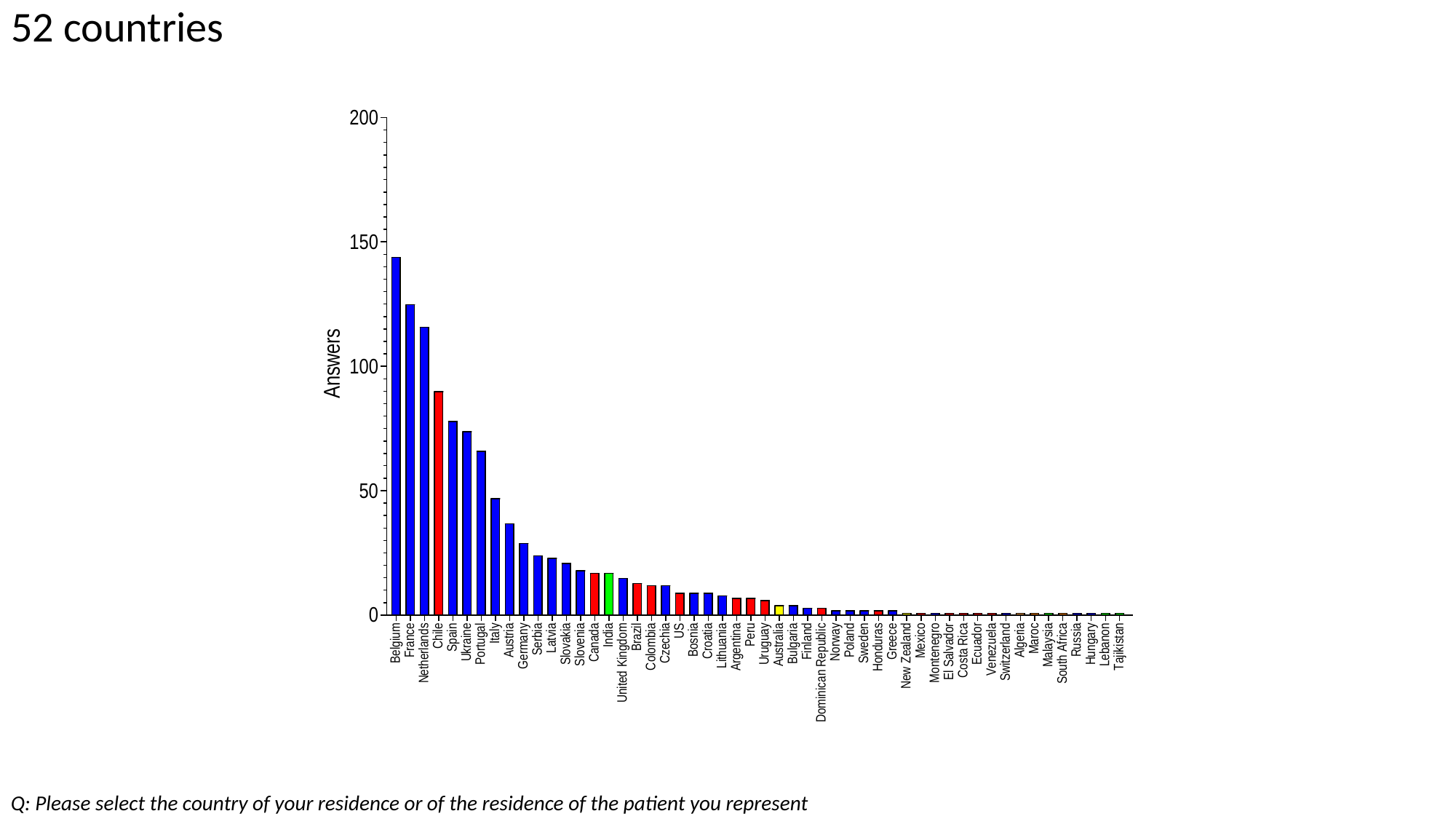

52 countries
Q: Please select the country of your residence or of the residence of the patient you represent

## Slide 3
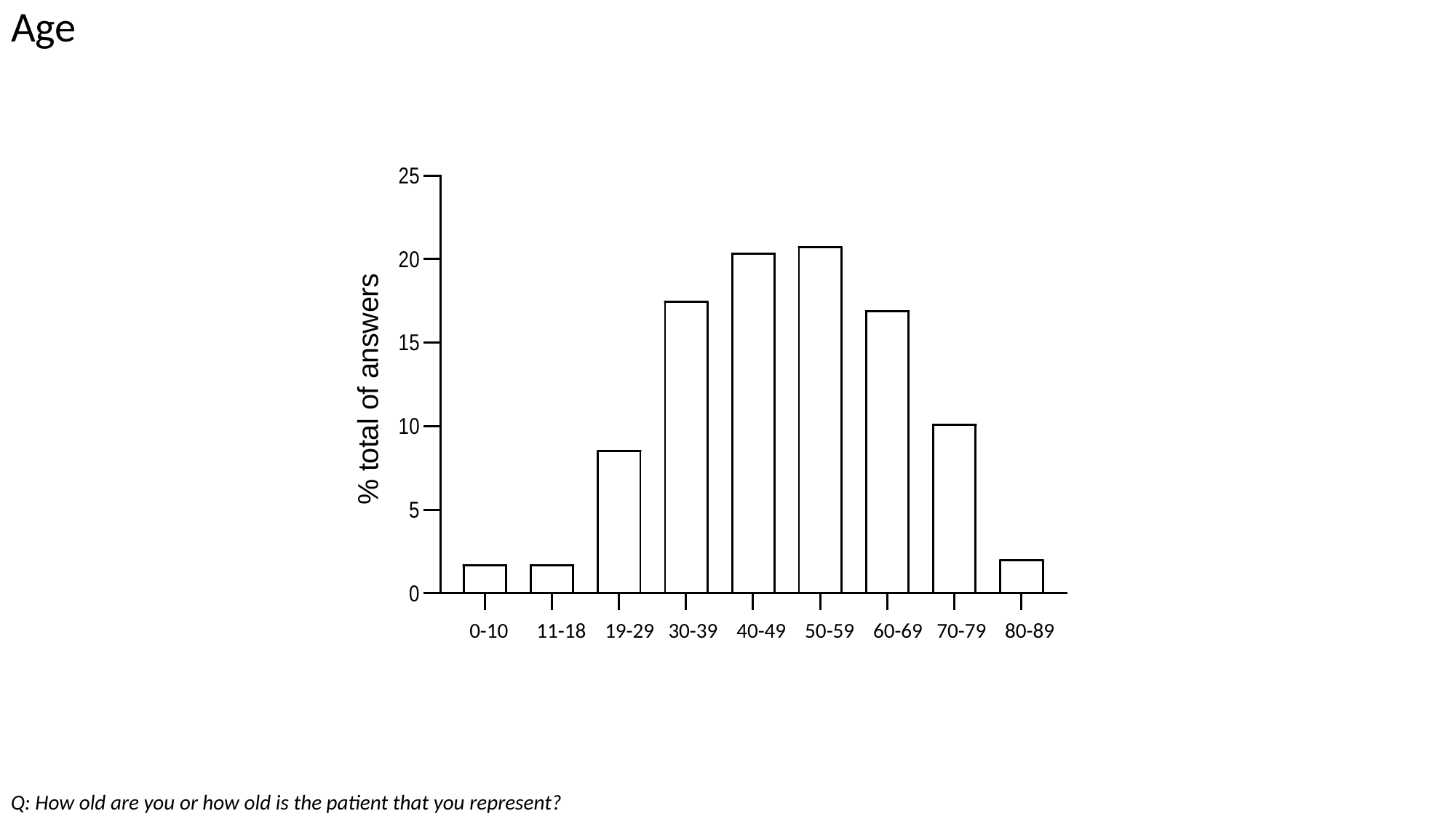

Age
 0-10 11-18 19-29 30-39 40-49 50-59 60-69 70-79 80-89
Q: How old are you or how old is the patient that you represent?

## Slide 4
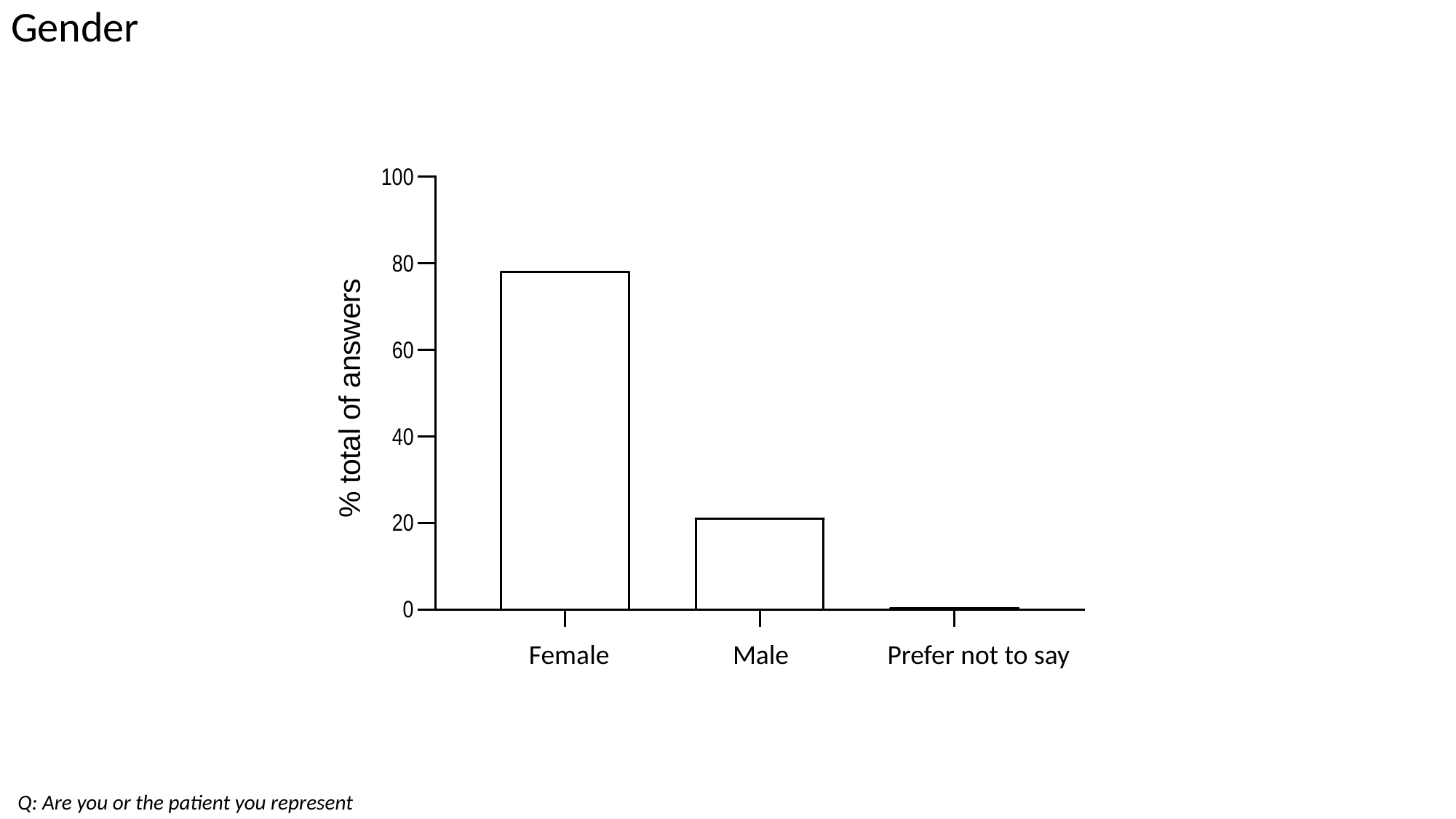

Gender
Female Male Prefer not to say
Q: Are you or the patient you represent

## Slide 5
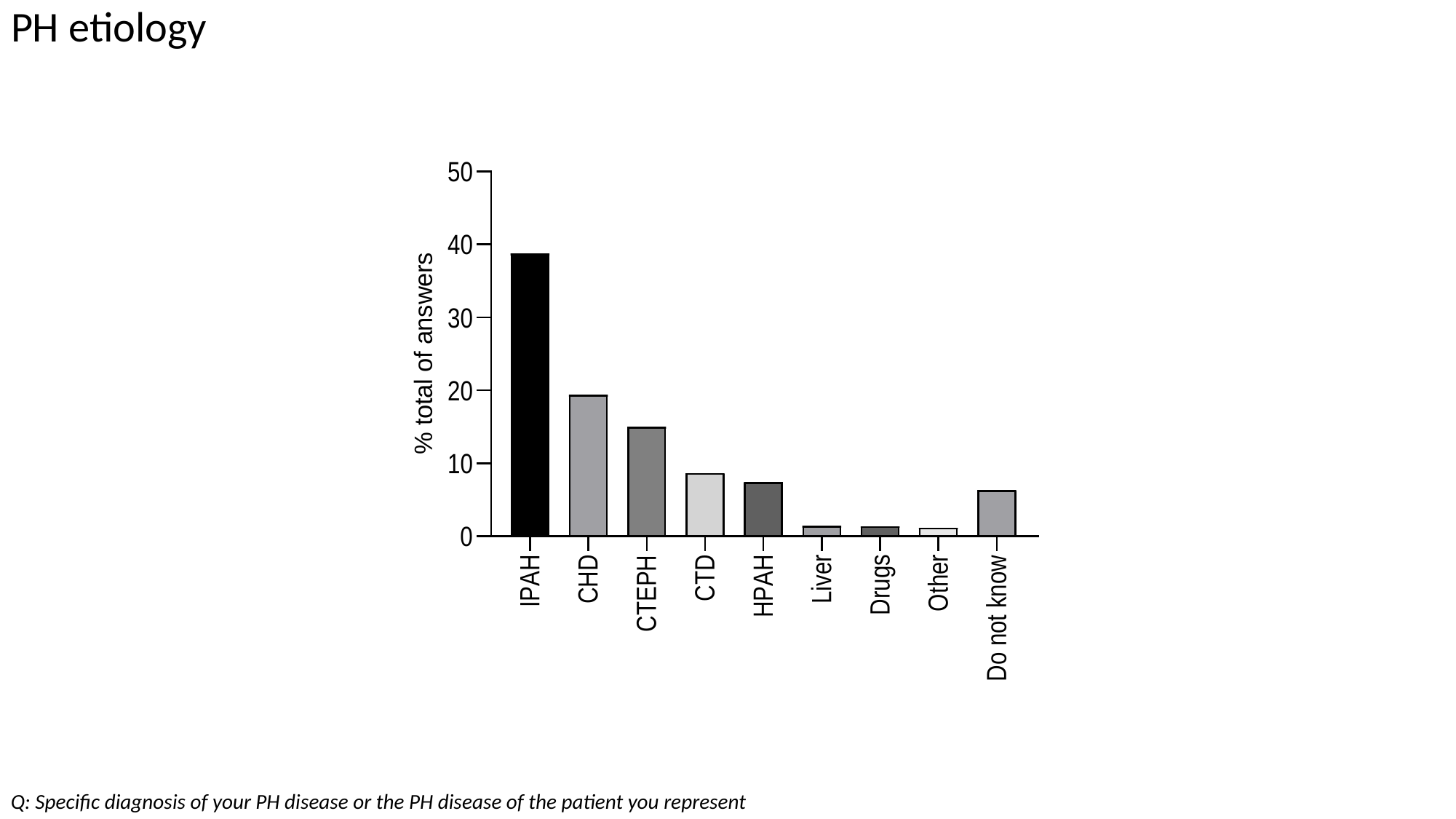

PH etiology
Q: Specific diagnosis of your PH disease or the PH disease of the patient you represent

## Slide 6
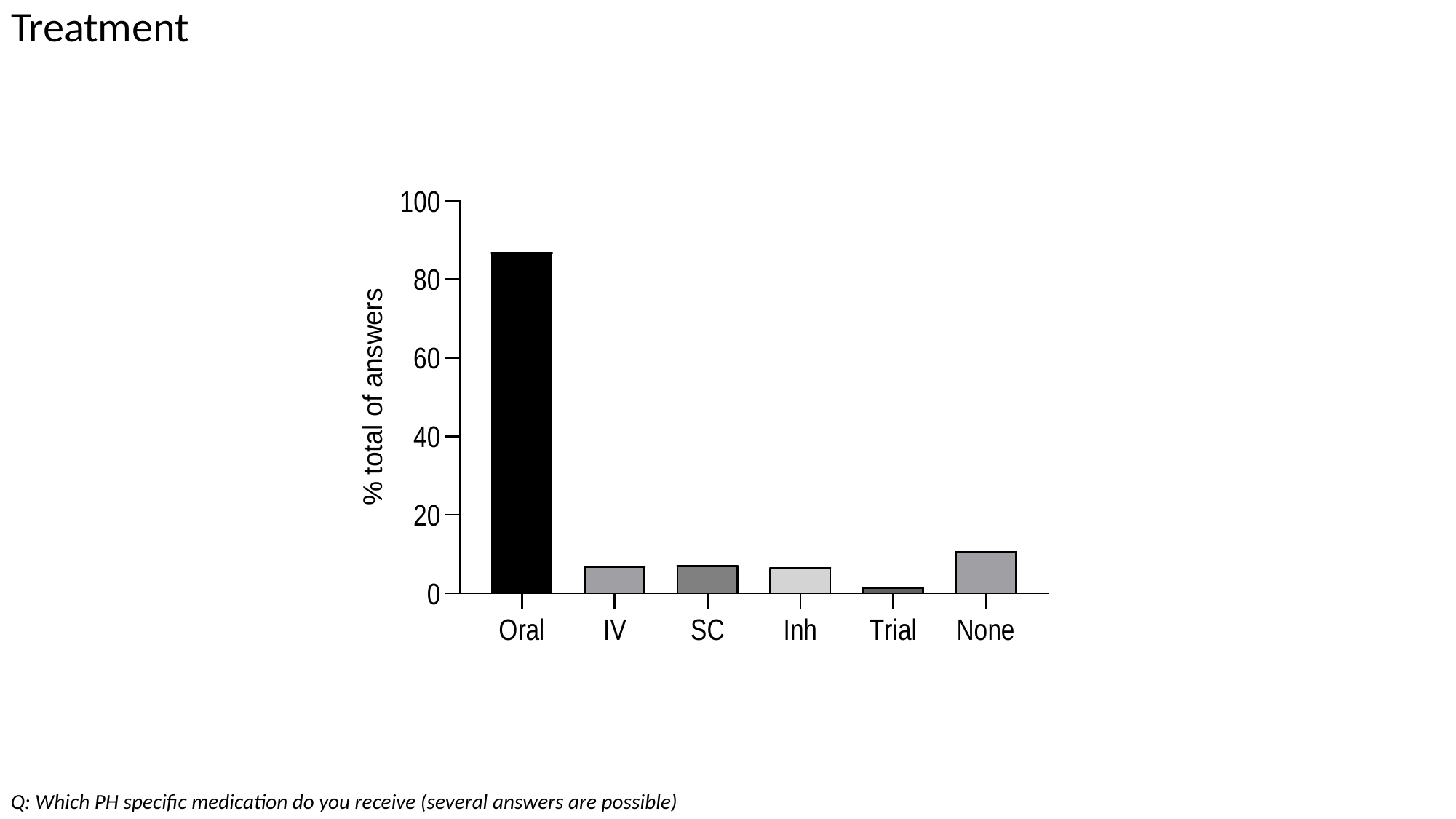

Treatment
Q: Which PH specific medication do you receive (several answers are possible)

## Slide 7
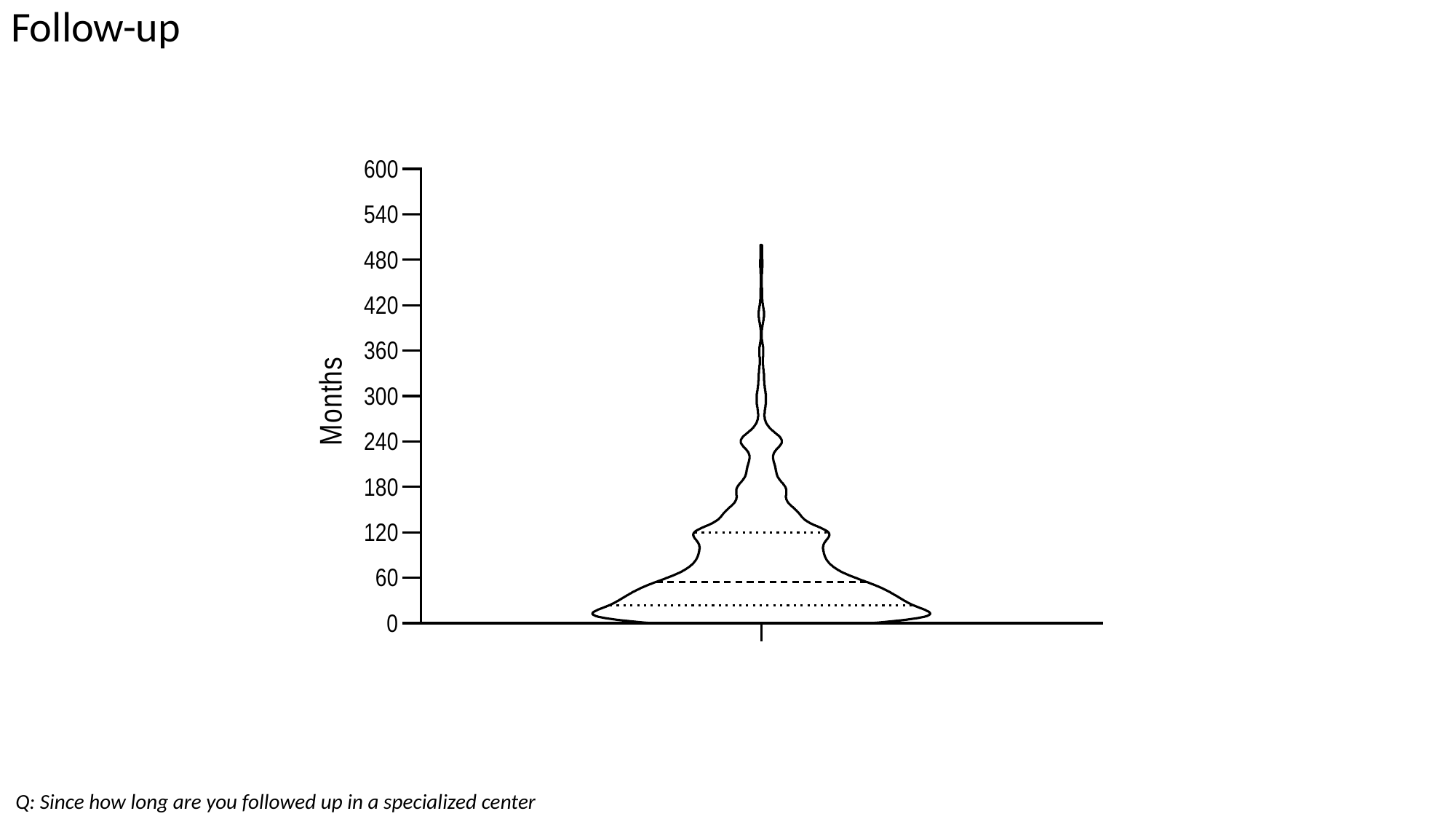

Follow-up
Q: Since how long are you followed up in a specialized center

## Slide 8
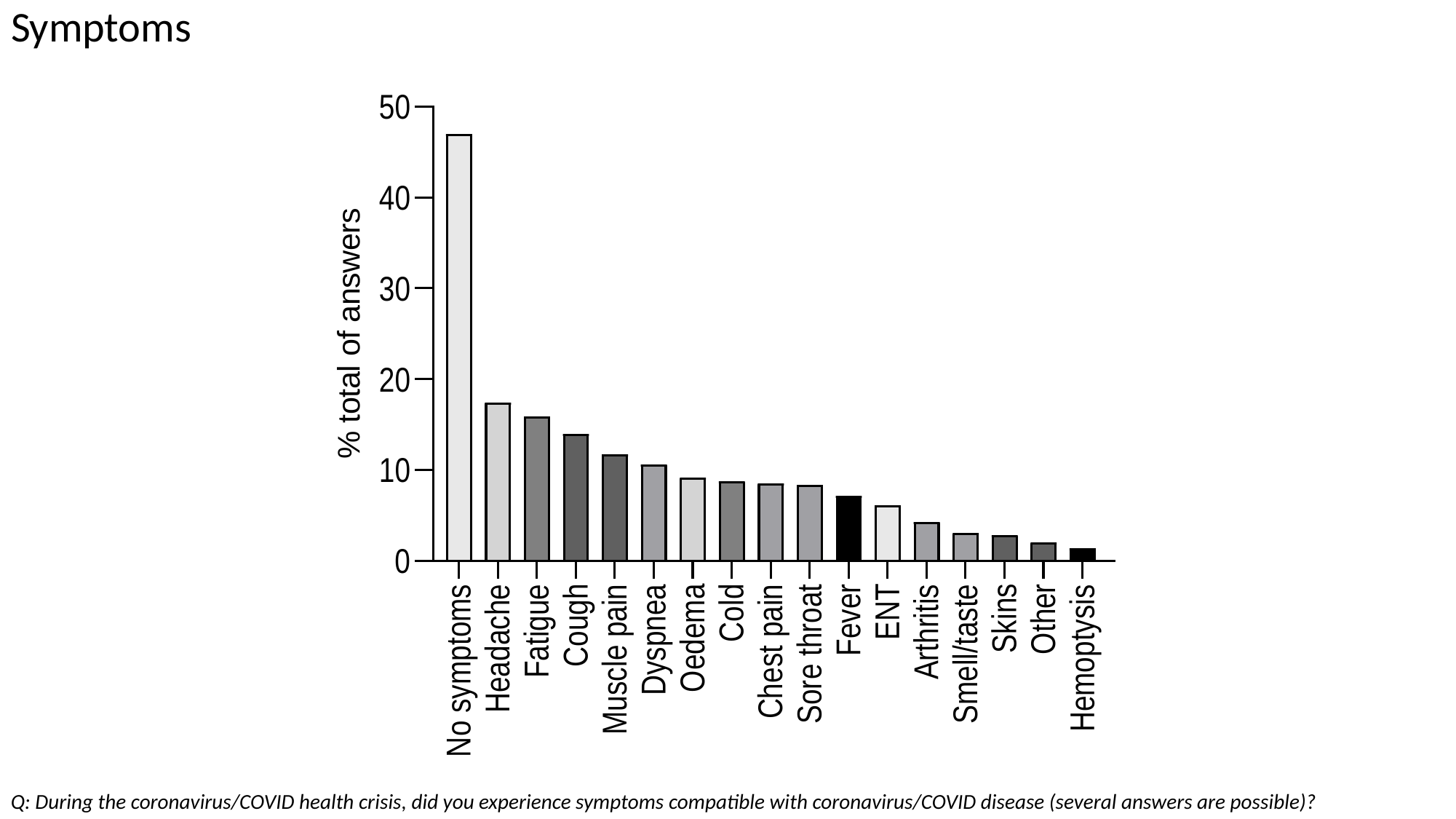

Symptoms
Q: During the coronavirus/COVID health crisis, did you experience symptoms compatible with coronavirus/COVID disease (several answers are possible)?

## Slide 9
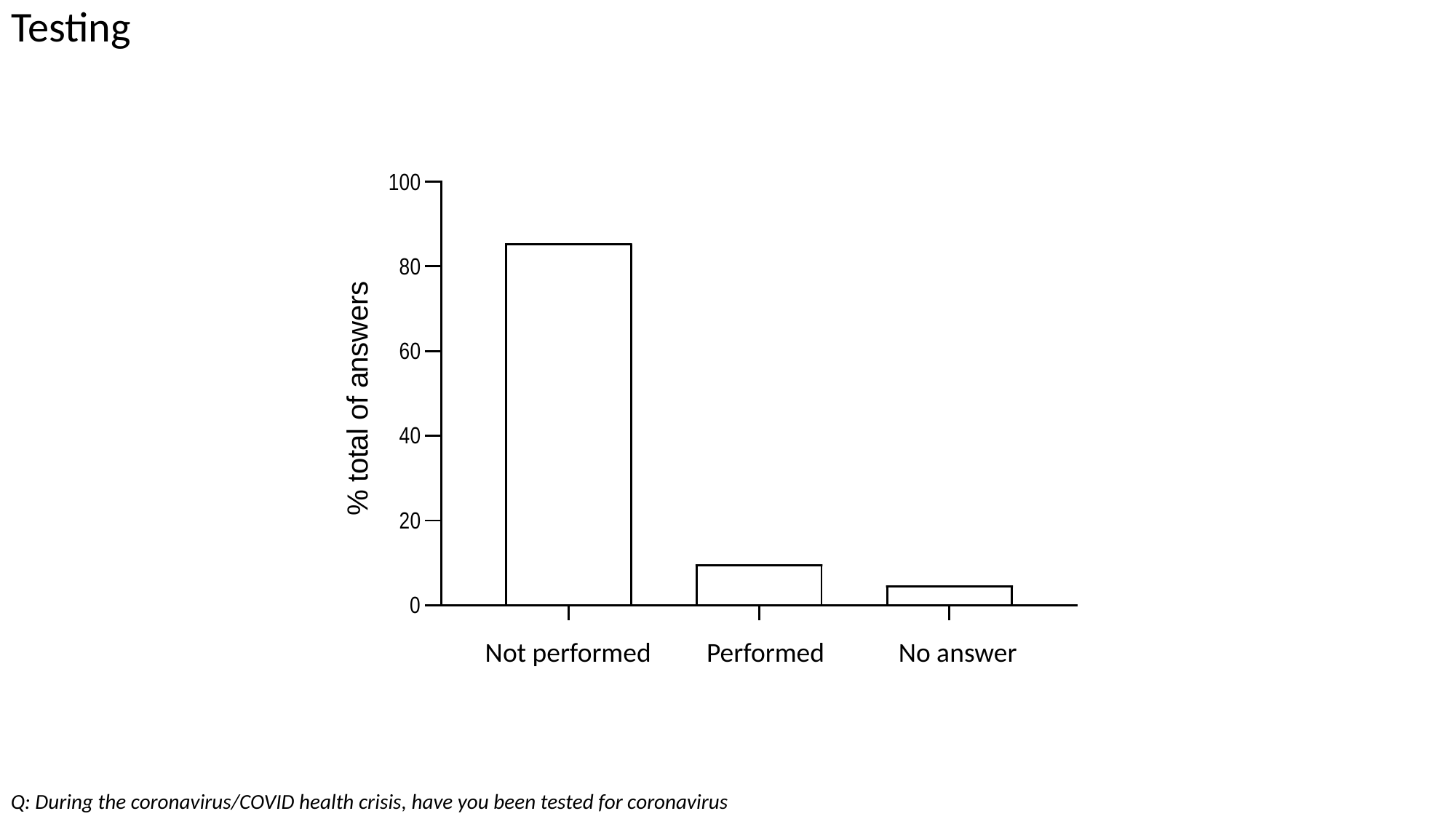

Testing
Not performed Performed No answer
Q: During the coronavirus/COVID health crisis, have you been tested for coronavirus

## Slide 10
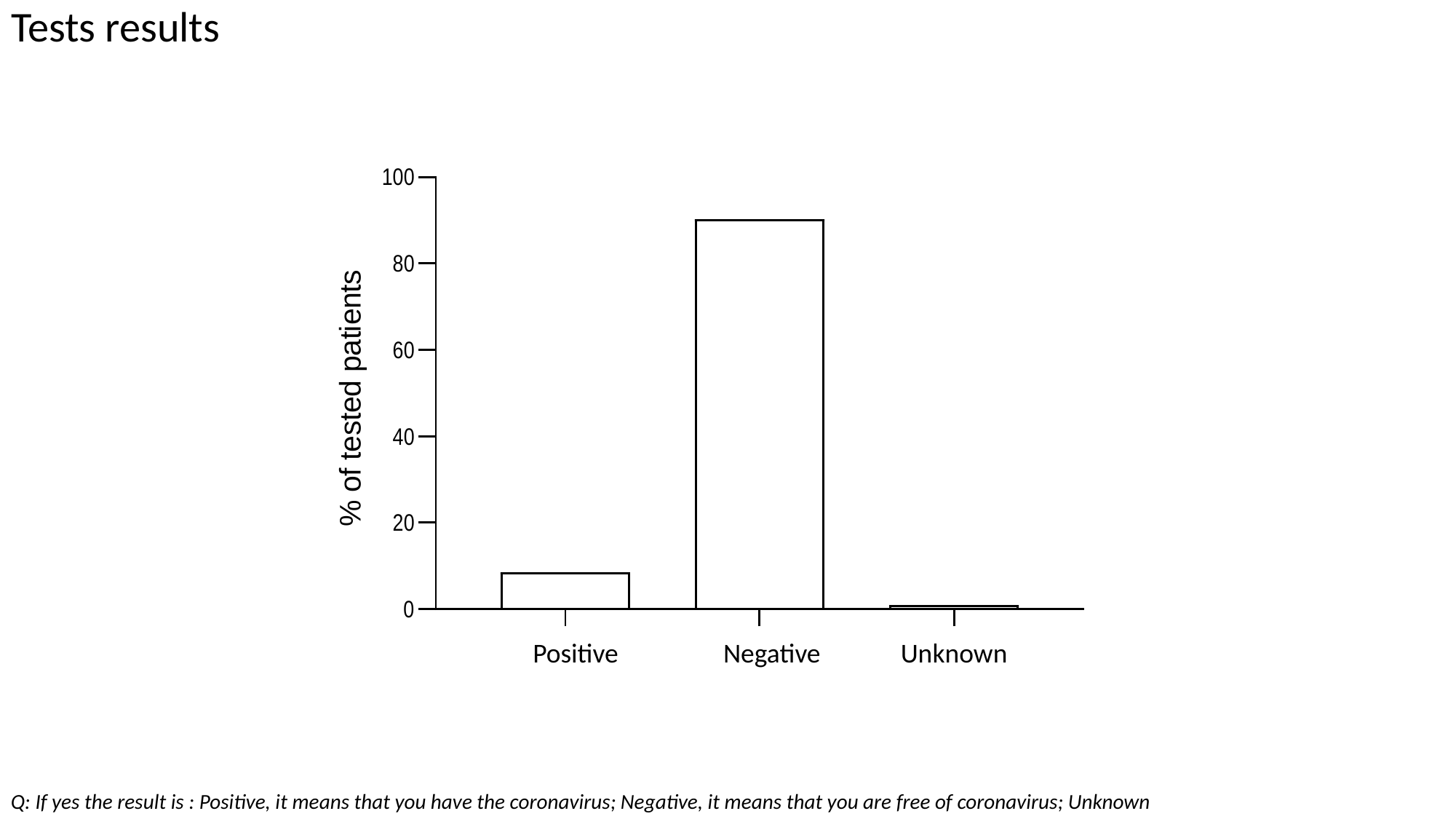

Tests results
 Positive Negative Unknown
Q: If yes the result is : Positive, it means that you have the coronavirus; Negative, it means that you are free of coronavirus; Unknown

## Slide 11
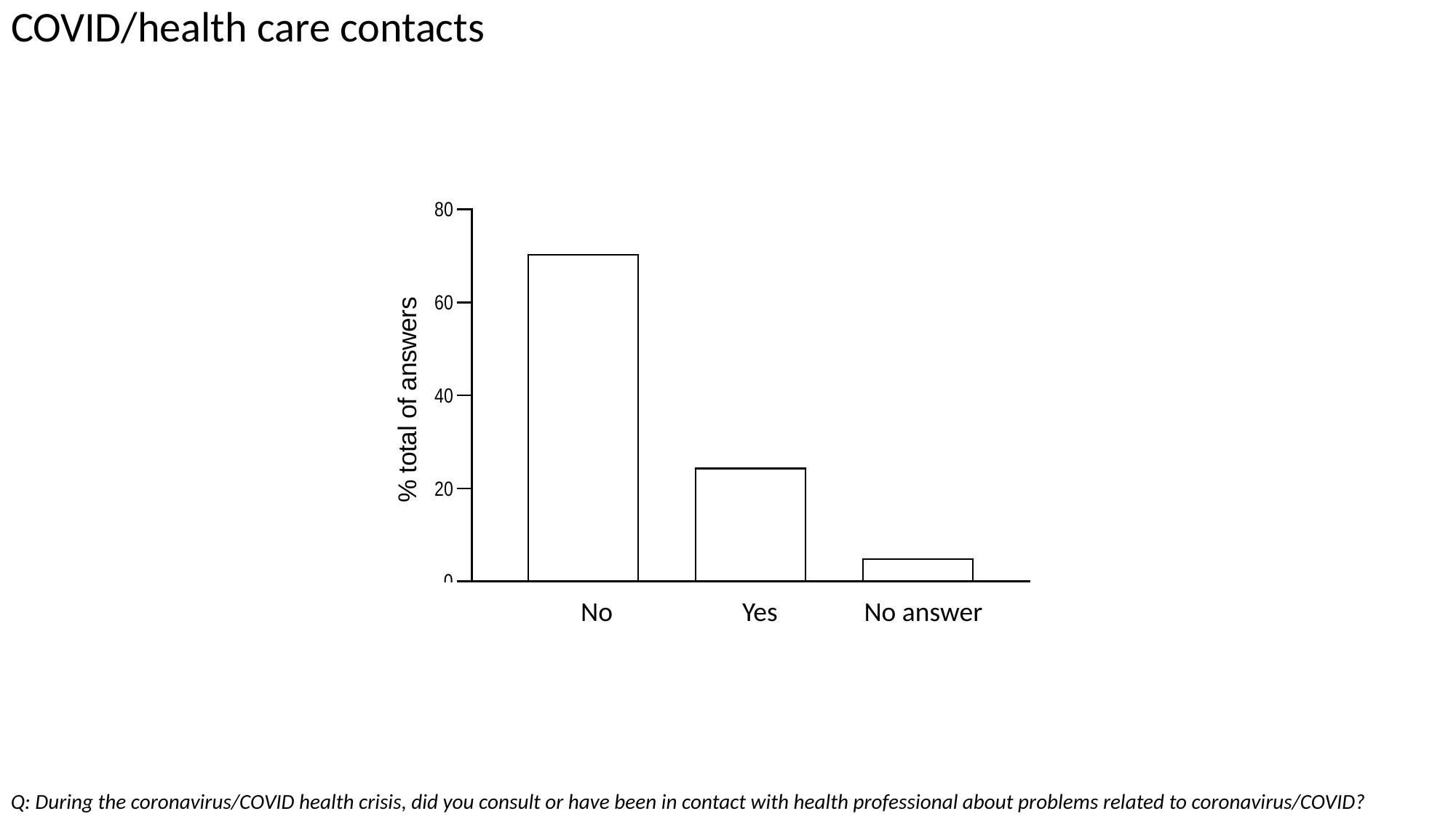

COVID/health care contacts
 No Yes No answer
Q: During the coronavirus/COVID health crisis, did you consult or have been in contact with health professional about problems related to coronavirus/COVID?

## Slide 12
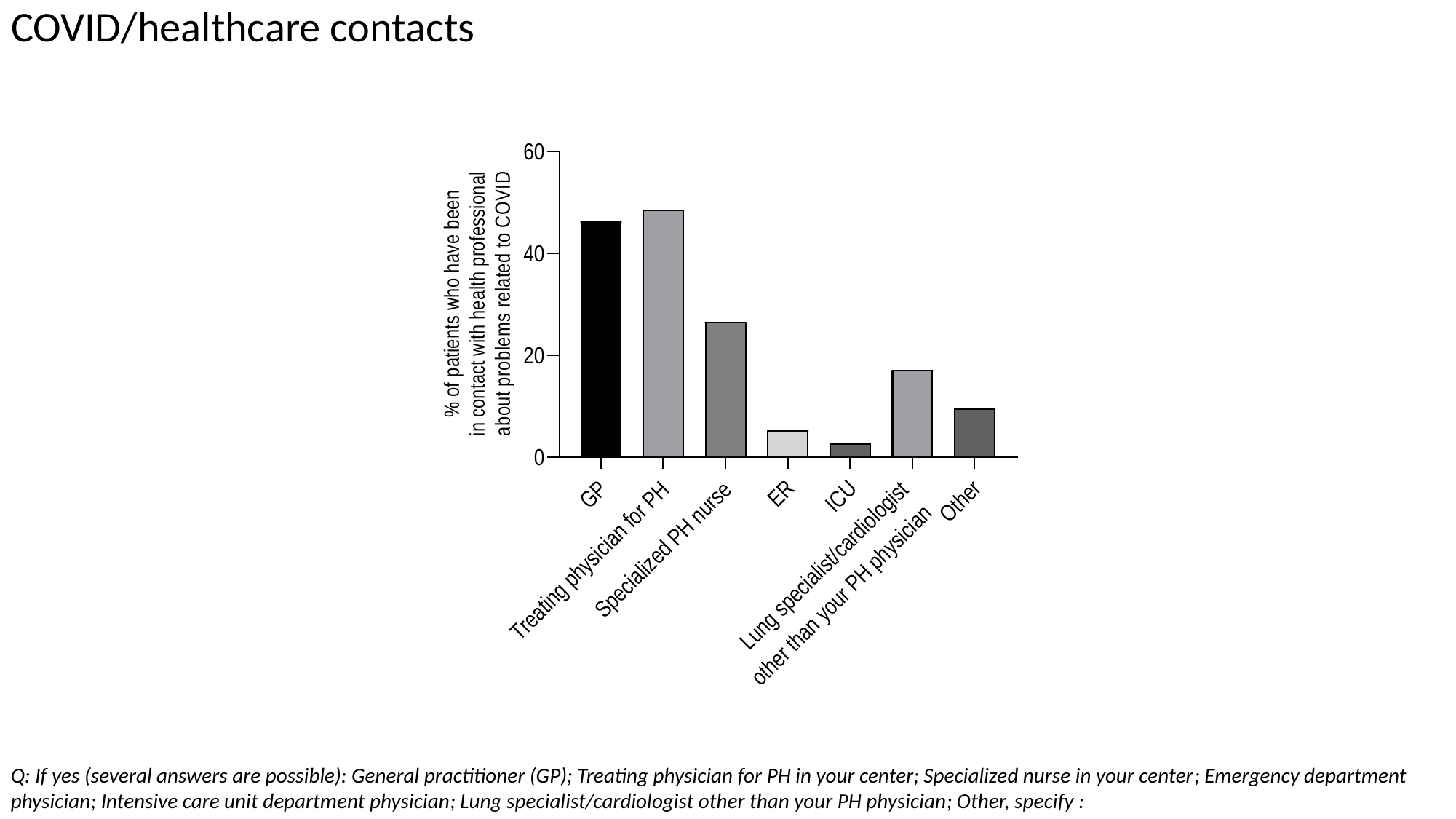

COVID/healthcare contacts
Q: If yes (several answers are possible): General practitioner (GP); Treating physician for PH in your center; Specialized nurse in your center; Emergency department physician; Intensive care unit department physician; Lung specialist/cardiologist other than your PH physician; Other, specify :

## Slide 13
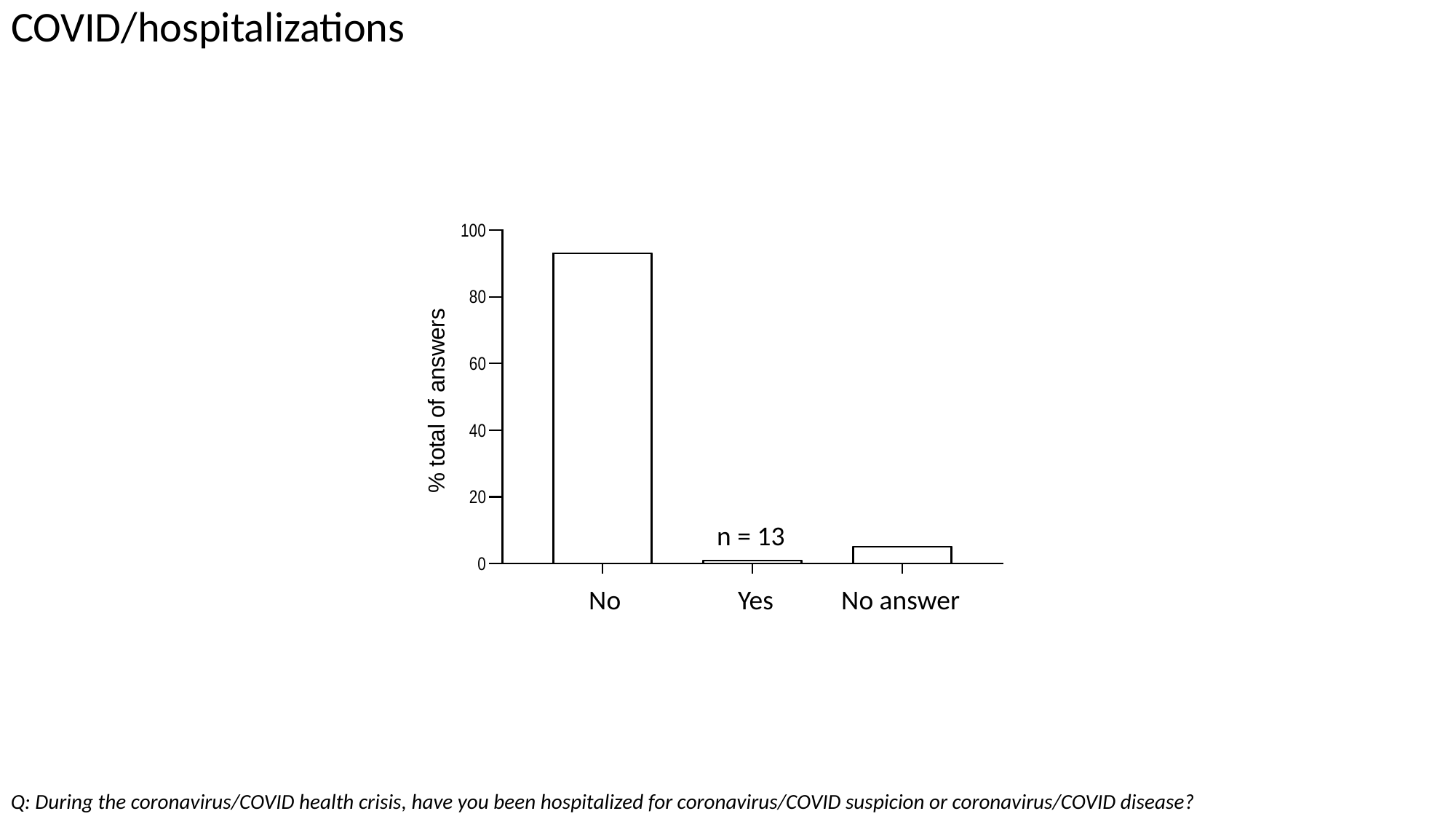

COVID/hospitalizations
n = 13
 No Yes No answer
Q: During the coronavirus/COVID health crisis, have you been hospitalized for coronavirus/COVID suspicion or coronavirus/COVID disease?

## Slide 14
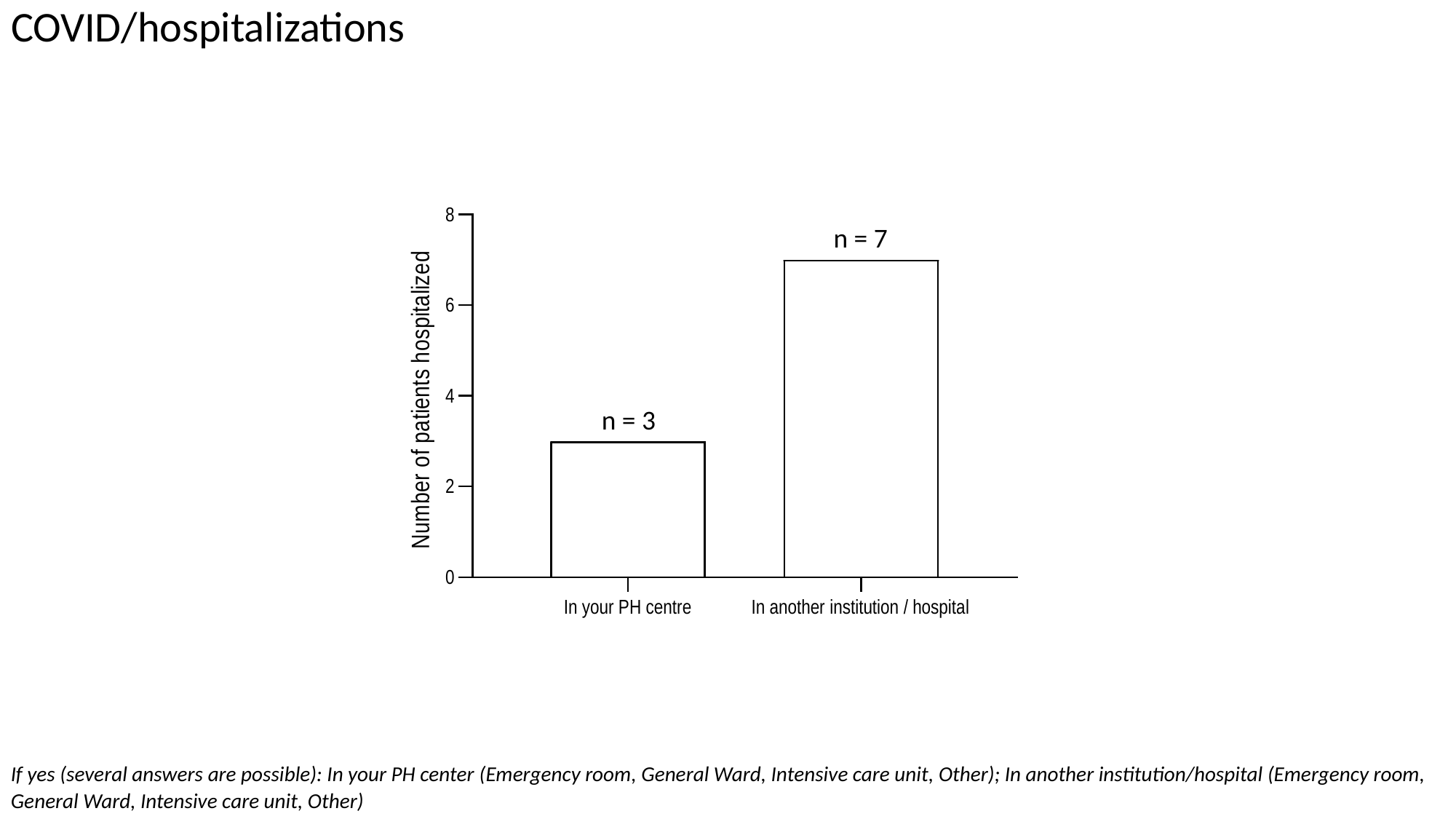

COVID/hospitalizations
n = 7
n = 3
If yes (several answers are possible): In your PH center (Emergency room, General Ward, Intensive care unit, Other); In another institution/hospital (Emergency room, General Ward, Intensive care unit, Other)

## Slide 15
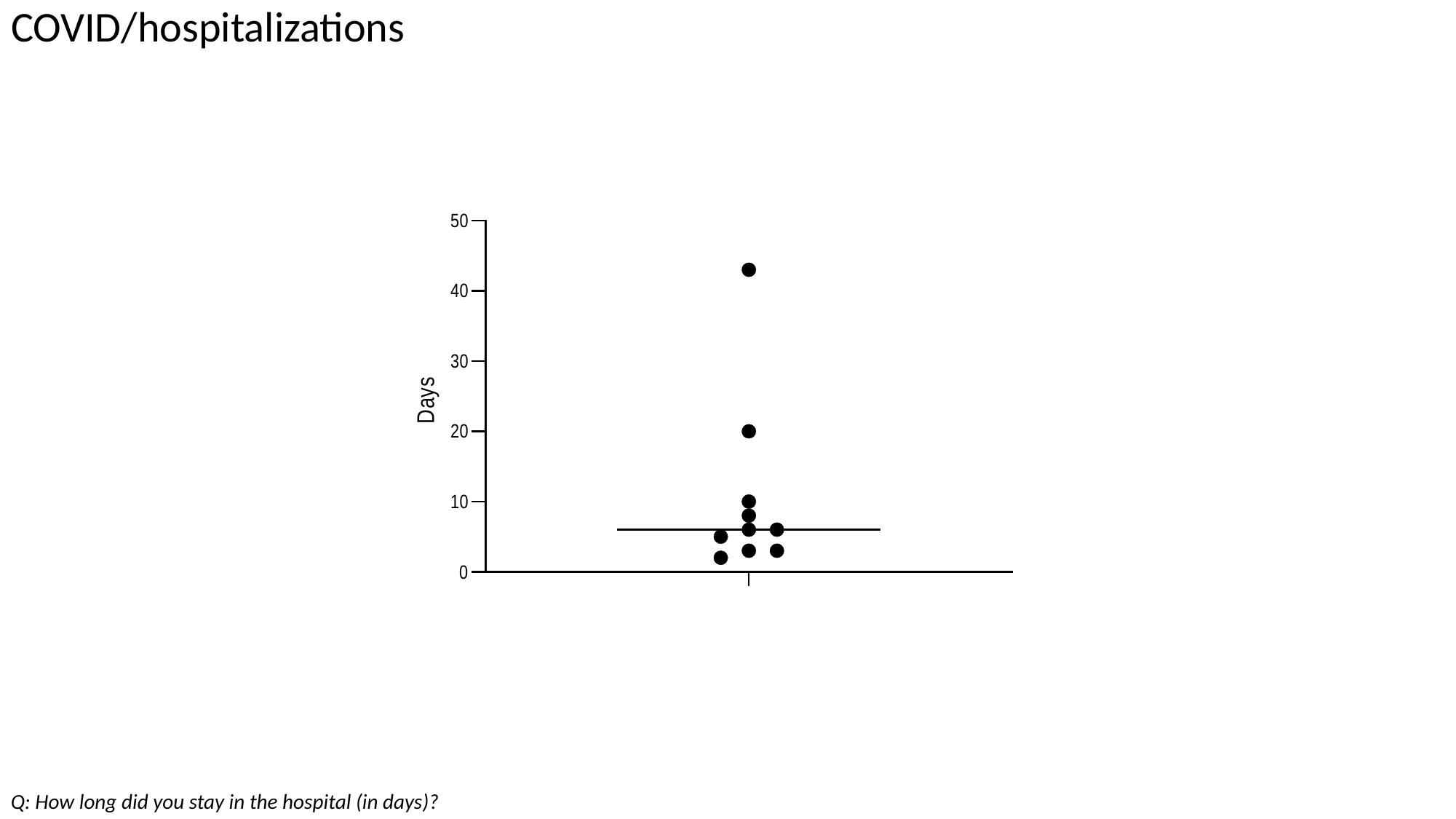

COVID/hospitalizations
Q: How long did you stay in the hospital (in days)?

## Slide 16
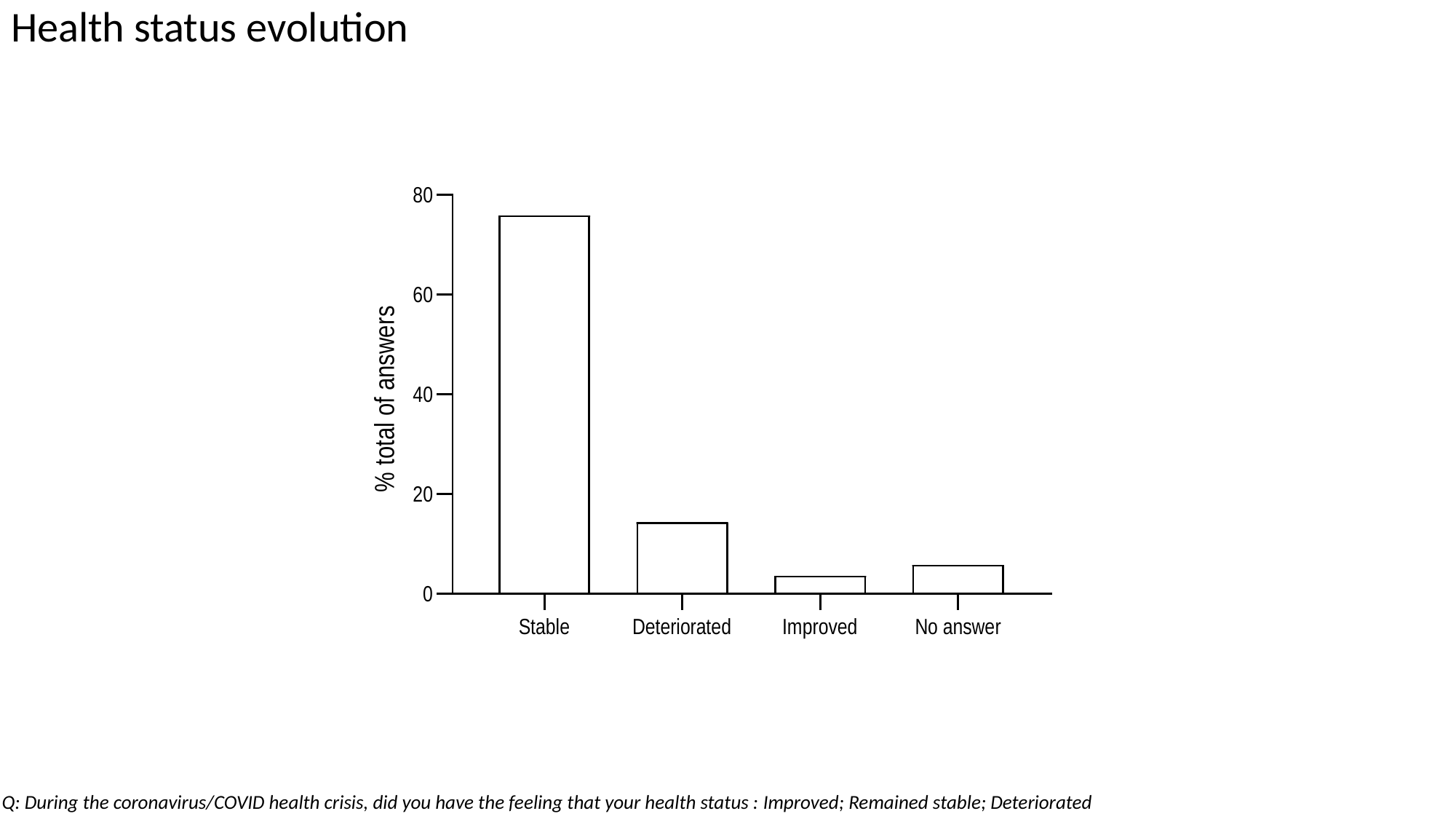

Health status evolution
Q: During the coronavirus/COVID health crisis, did you have the feeling that your health status : Improved; Remained stable; Deteriorated

## Slide 17
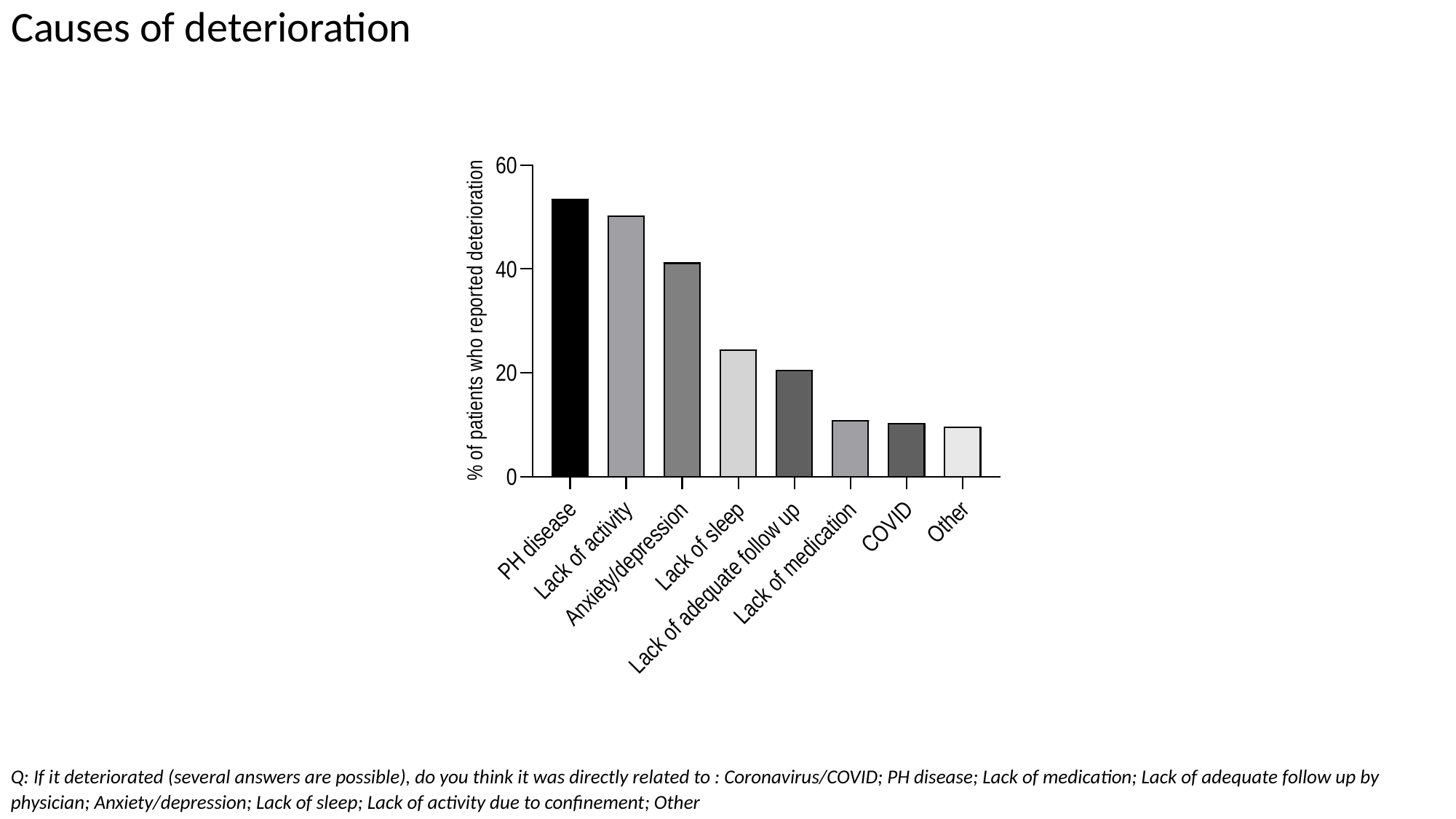

Causes of deterioration
Q: If it deteriorated (several answers are possible), do you think it was directly related to : Coronavirus/COVID; PH disease; Lack of medication; Lack of adequate follow up by physician; Anxiety/depression; Lack of sleep; Lack of activity due to confinement; Other

## Slide 18
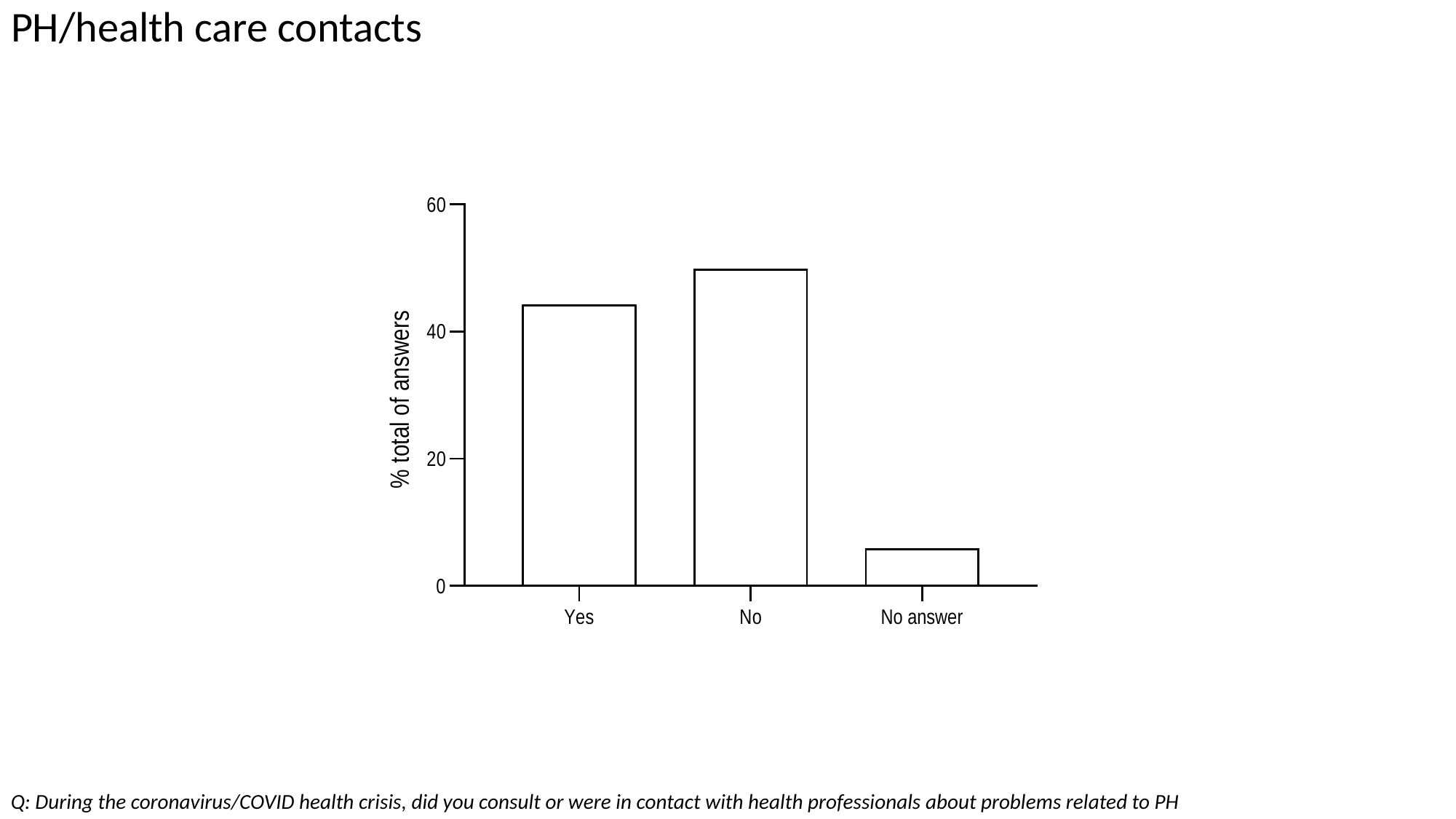

PH/health care contacts
Q: During the coronavirus/COVID health crisis, did you consult or were in contact with health professionals about problems related to PH

## Slide 19
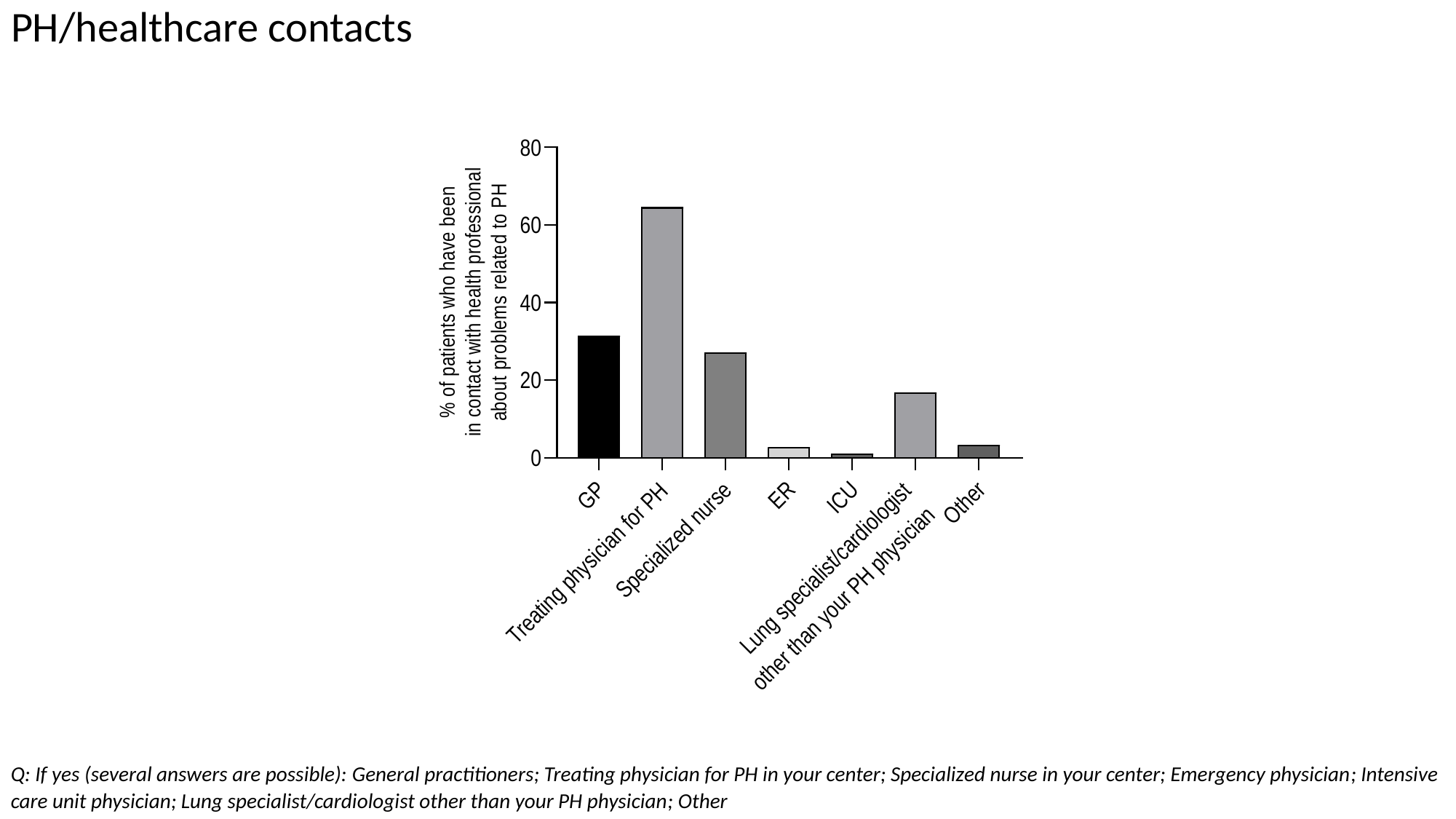

PH/healthcare contacts
Q: If yes (several answers are possible): General practitioners; Treating physician for PH in your center; Specialized nurse in your center; Emergency physician; Intensive care unit physician; Lung specialist/cardiologist other than your PH physician; Other

## Slide 20
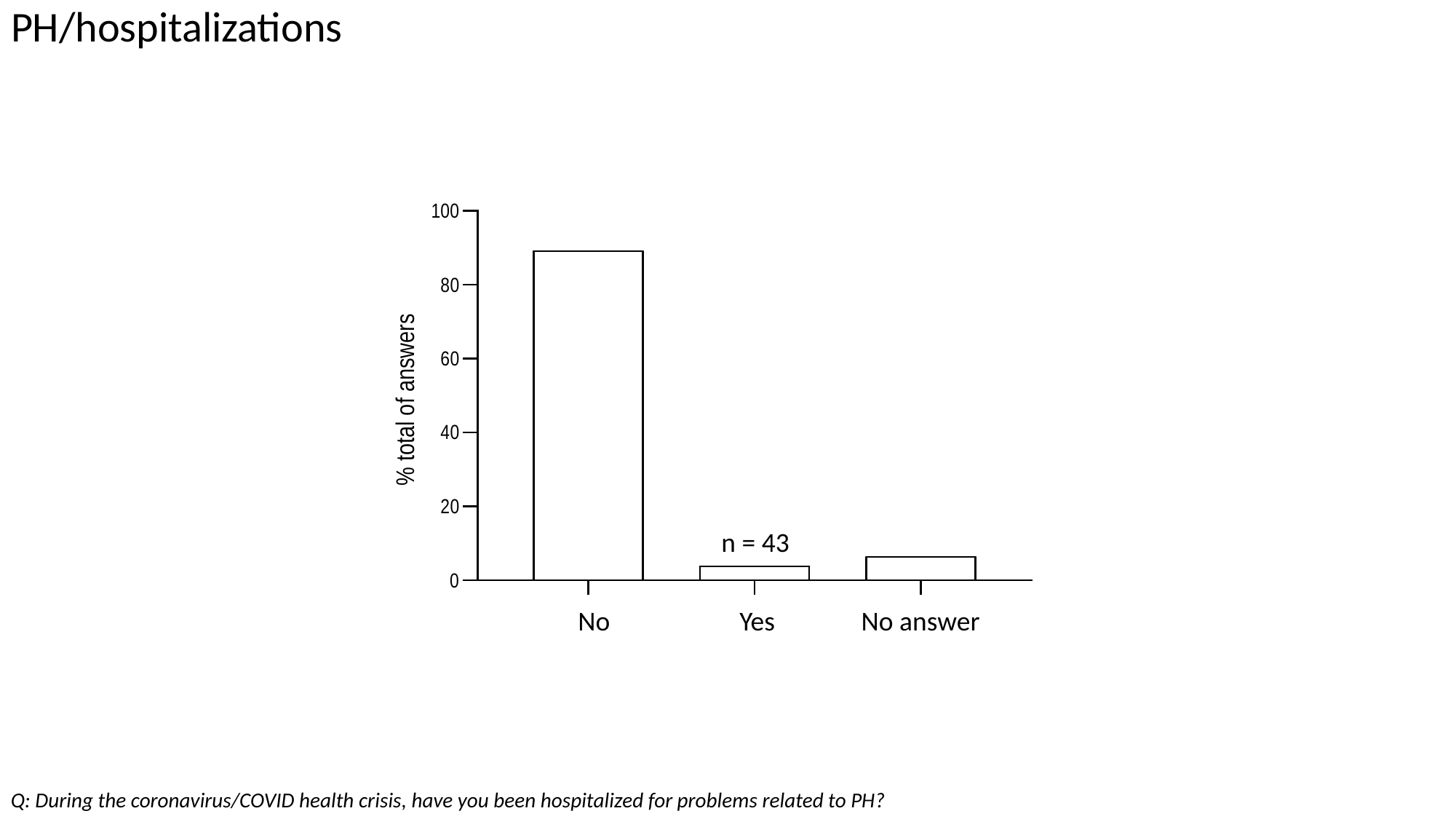

PH/hospitalizations
n = 43
 No Yes No answer
Q: During the coronavirus/COVID health crisis, have you been hospitalized for problems related to PH?

## Slide 21
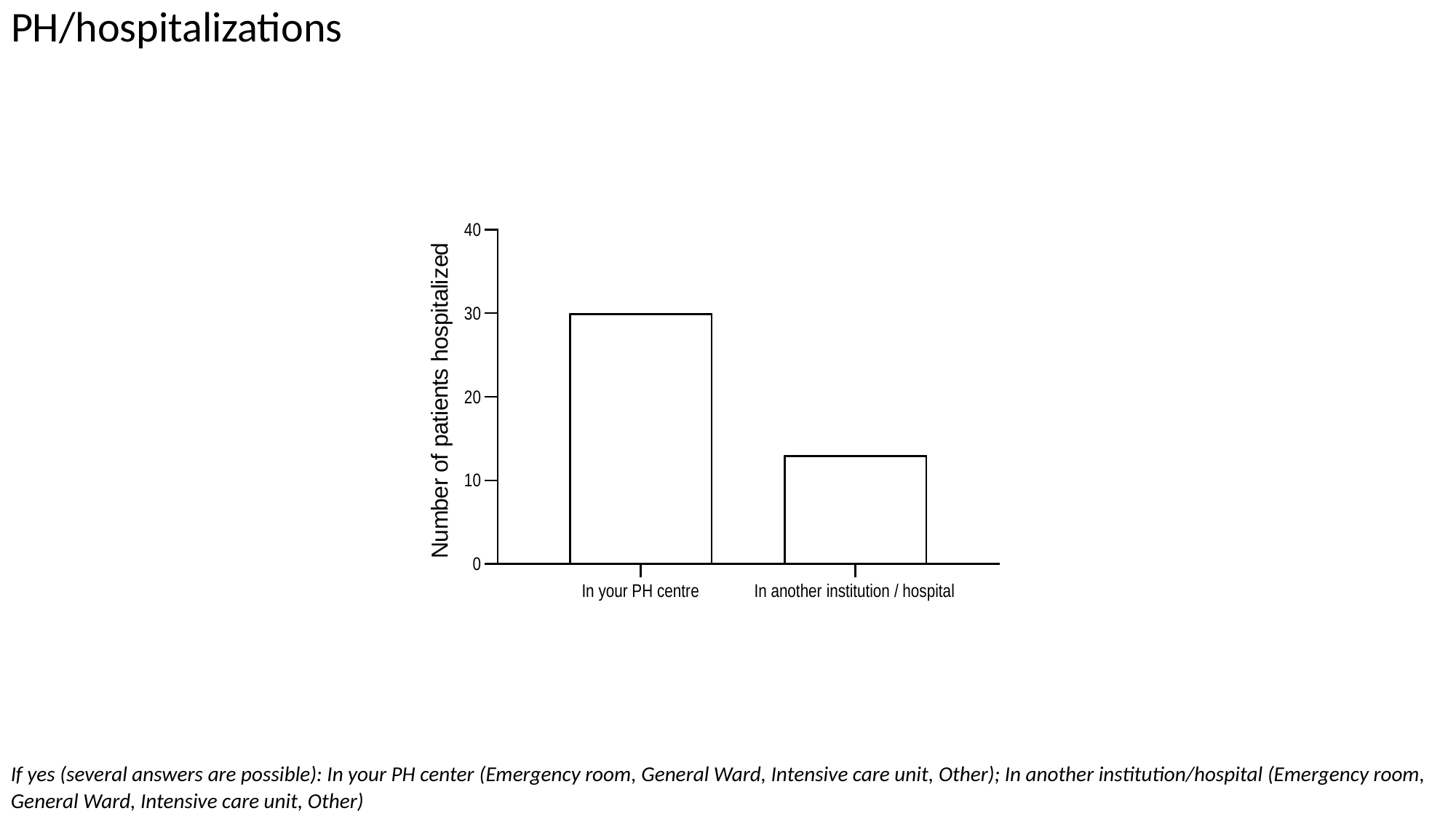

PH/hospitalizations
If yes (several answers are possible): In your PH center (Emergency room, General Ward, Intensive care unit, Other); In another institution/hospital (Emergency room, General Ward, Intensive care unit, Other)

## Slide 22
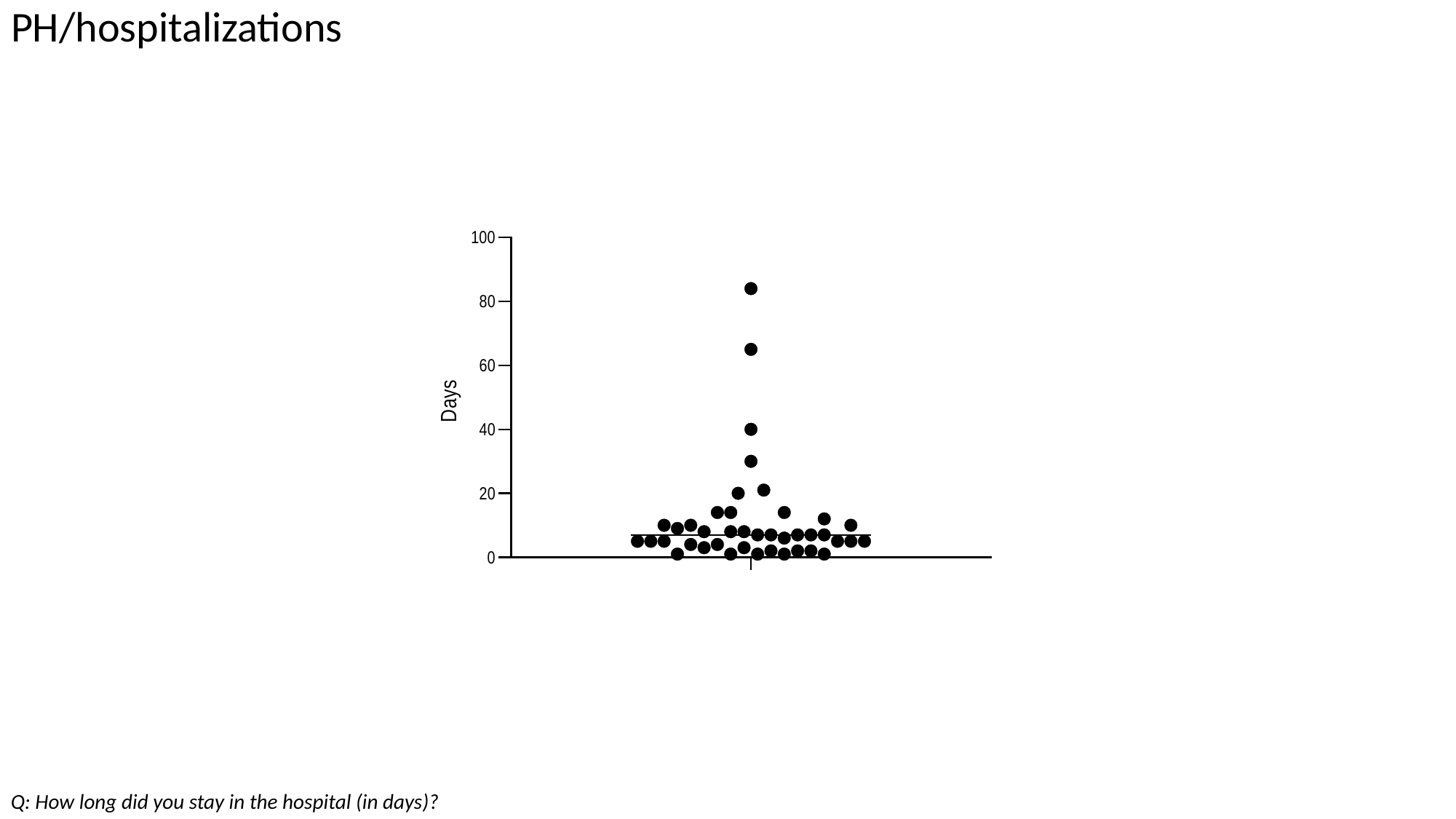

PH/hospitalizations
Q: How long did you stay in the hospital (in days)?

## Slide 23
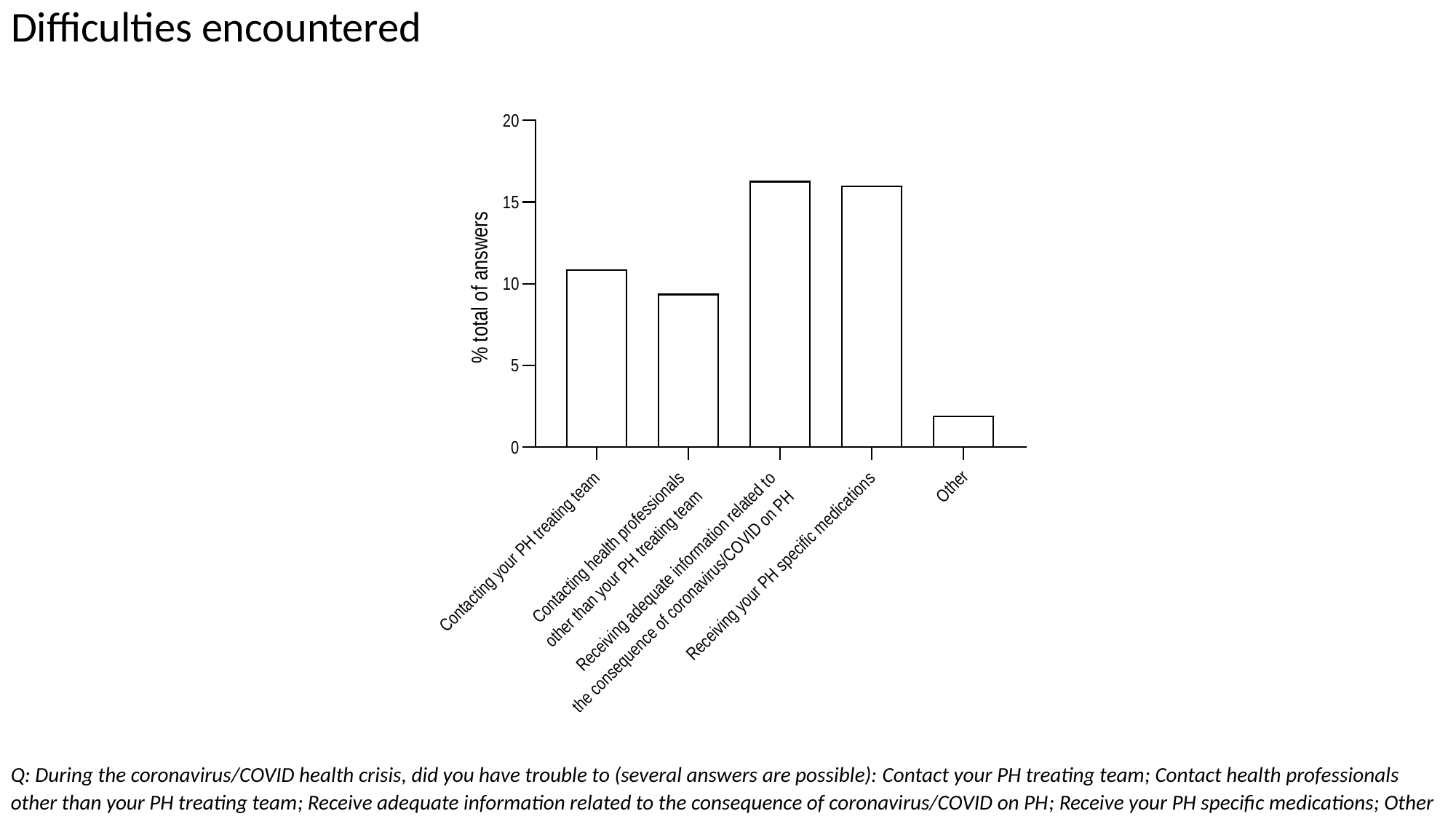

Difficulties encountered
Q: During the coronavirus/COVID health crisis, did you have trouble to (several answers are possible): Contact your PH treating team; Contact health professionals other than your PH treating team; Receive adequate information related to the consequence of coronavirus/COVID on PH; Receive your PH specific medications; Other

## Slide 24
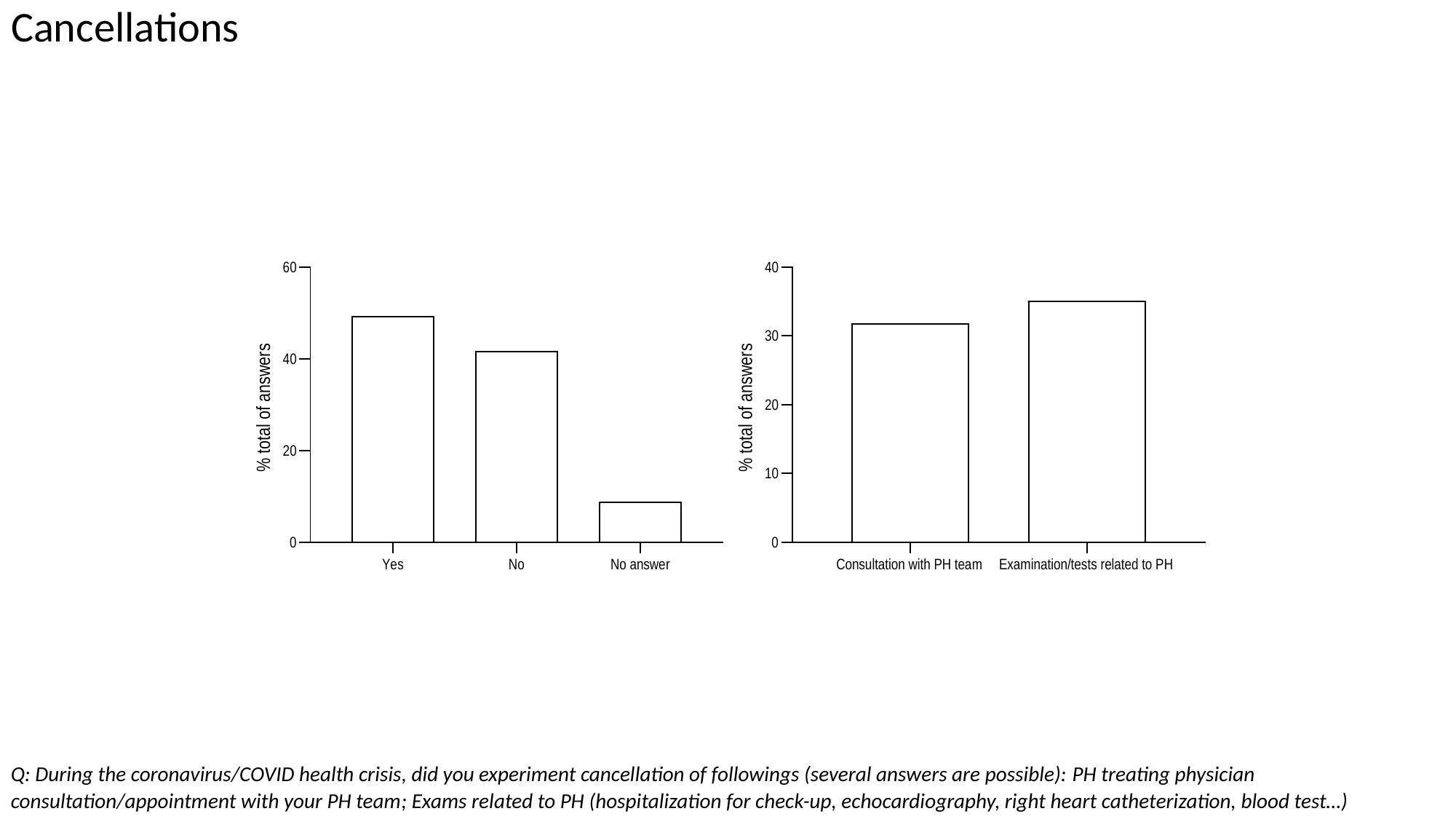

Cancellations
Q: During the coronavirus/COVID health crisis, did you experiment cancellation of followings (several answers are possible): PH treating physician consultation/appointment with your PH team; Exams related to PH (hospitalization for check-up, echocardiography, right heart catheterization, blood test…)

## Slide 25
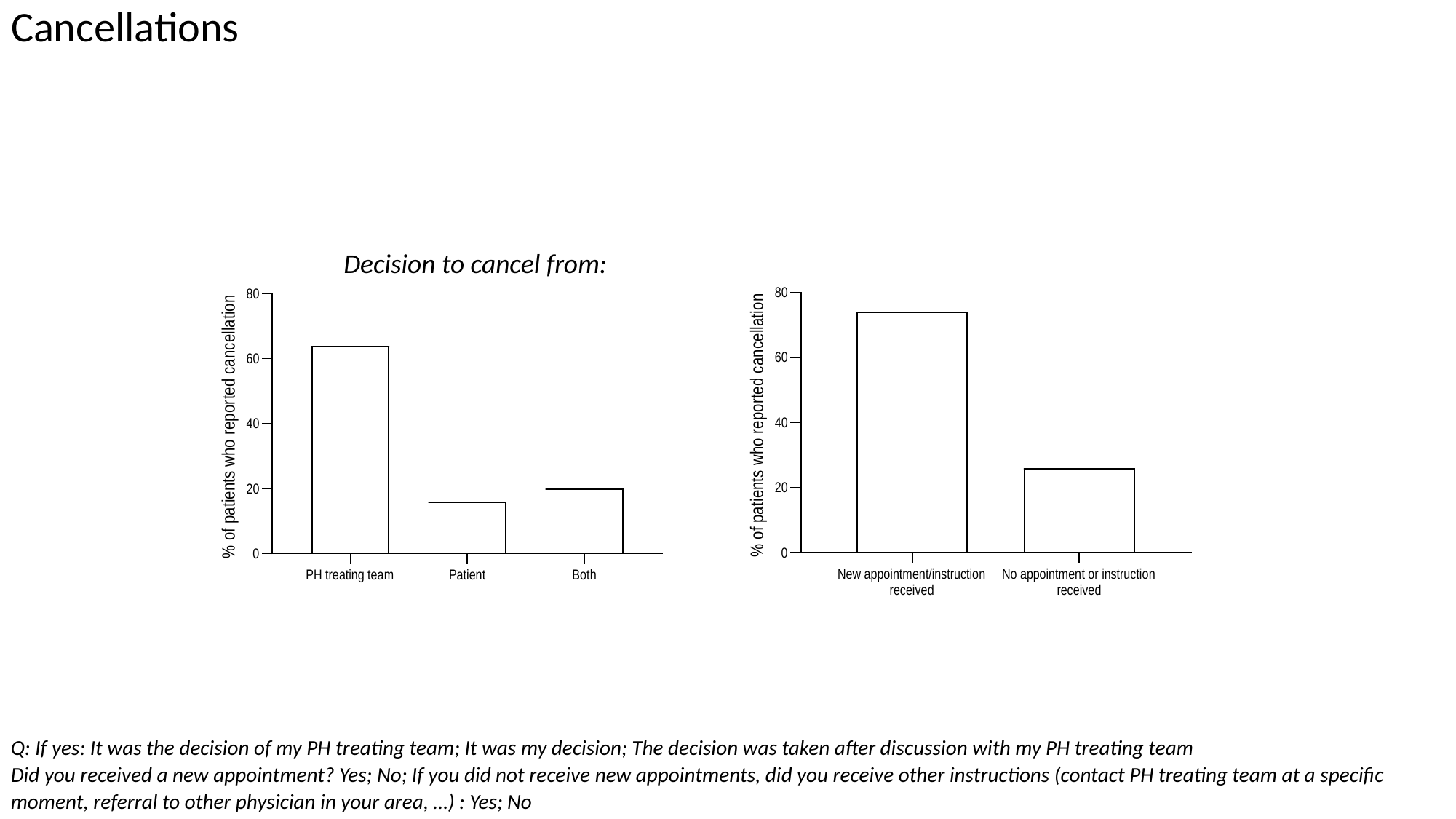

Cancellations
Decision to cancel from:
Q: If yes: It was the decision of my PH treating team; It was my decision; The decision was taken after discussion with my PH treating team
Did you received a new appointment? Yes; No; If you did not receive new appointments, did you receive other instructions (contact PH treating team at a specific moment, referral to other physician in your area, …) : Yes; No

## Slide 26
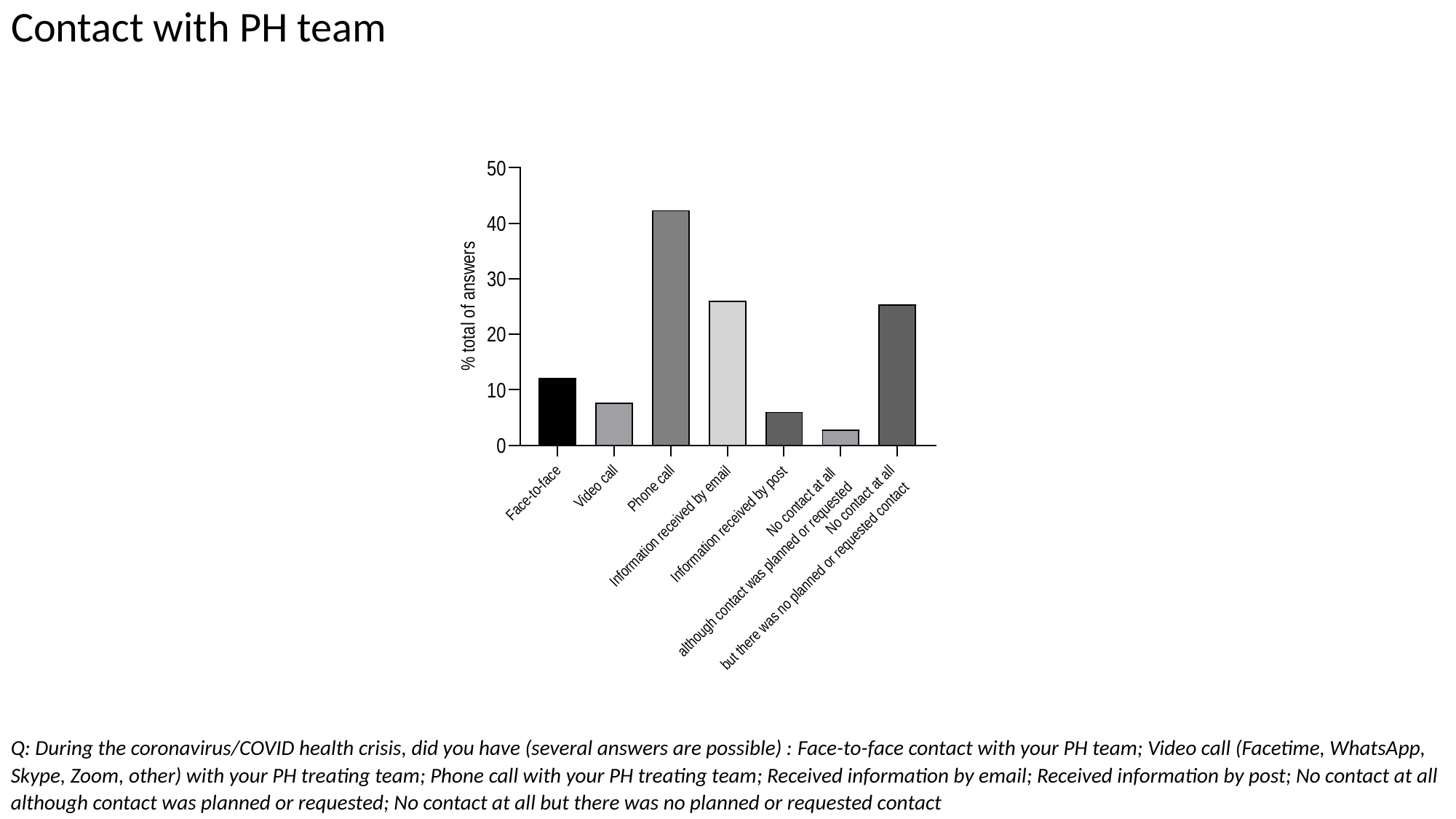

Contact with PH team
Q: During the coronavirus/COVID health crisis, did you have (several answers are possible) : Face-to-face contact with your PH team; Video call (Facetime, WhatsApp, Skype, Zoom, other) with your PH treating team; Phone call with your PH treating team; Received information by email; Received information by post; No contact at all although contact was planned or requested; No contact at all but there was no planned or requested contact

## Slide 27
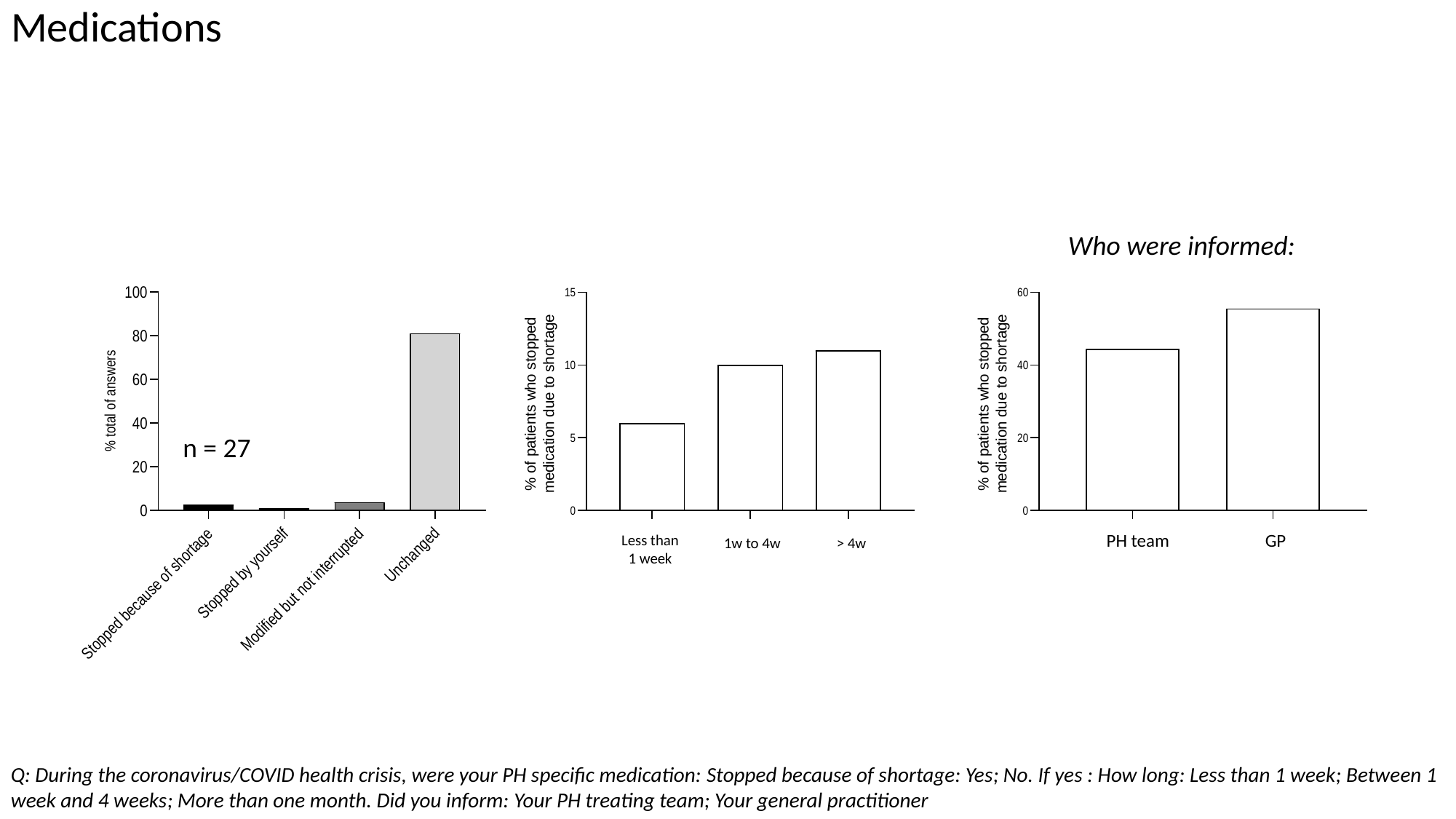

Medications
Who were informed:
n = 27
 PH team GP
Less than
1 week
1w to 4w
> 4w
Q: During the coronavirus/COVID health crisis, were your PH specific medication: Stopped because of shortage: Yes; No. If yes : How long: Less than 1 week; Between 1 week and 4 weeks; More than one month. Did you inform: Your PH treating team; Your general practitioner

## Slide 28
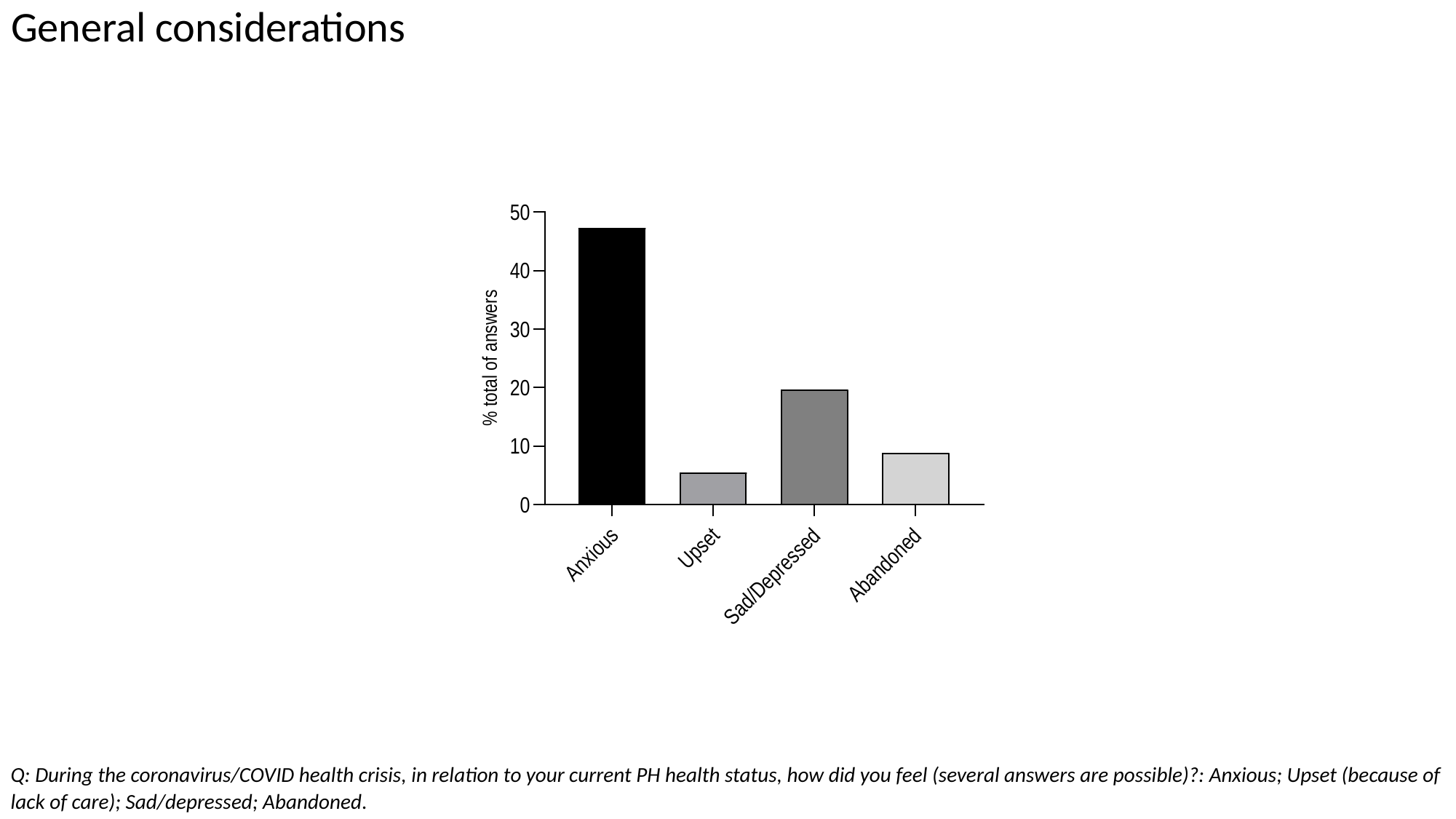

General considerations
Q: During the coronavirus/COVID health crisis, in relation to your current PH health status, how did you feel (several answers are possible)?: Anxious; Upset (because of lack of care); Sad/depressed; Abandoned.

## Slide 29
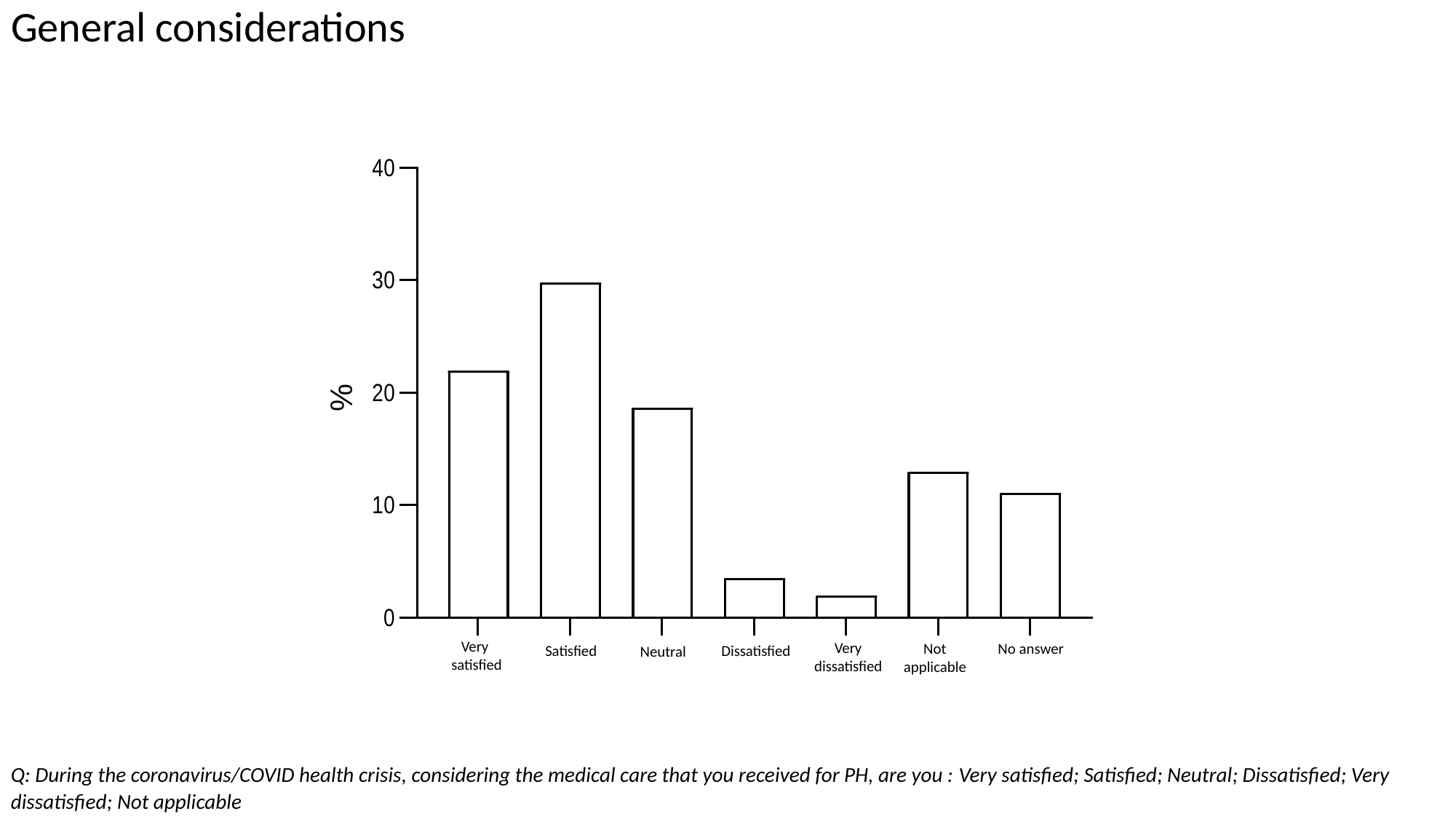

General considerations
Neutral
Very
satisfied
Very
dissatisfied
Not
applicable
No answer
Dissatisfied
 Satisfied
Q: During the coronavirus/COVID health crisis, considering the medical care that you received for PH, are you : Very satisfied; Satisfied; Neutral; Dissatisfied; Very dissatisfied; Not applicable

## Slide 30
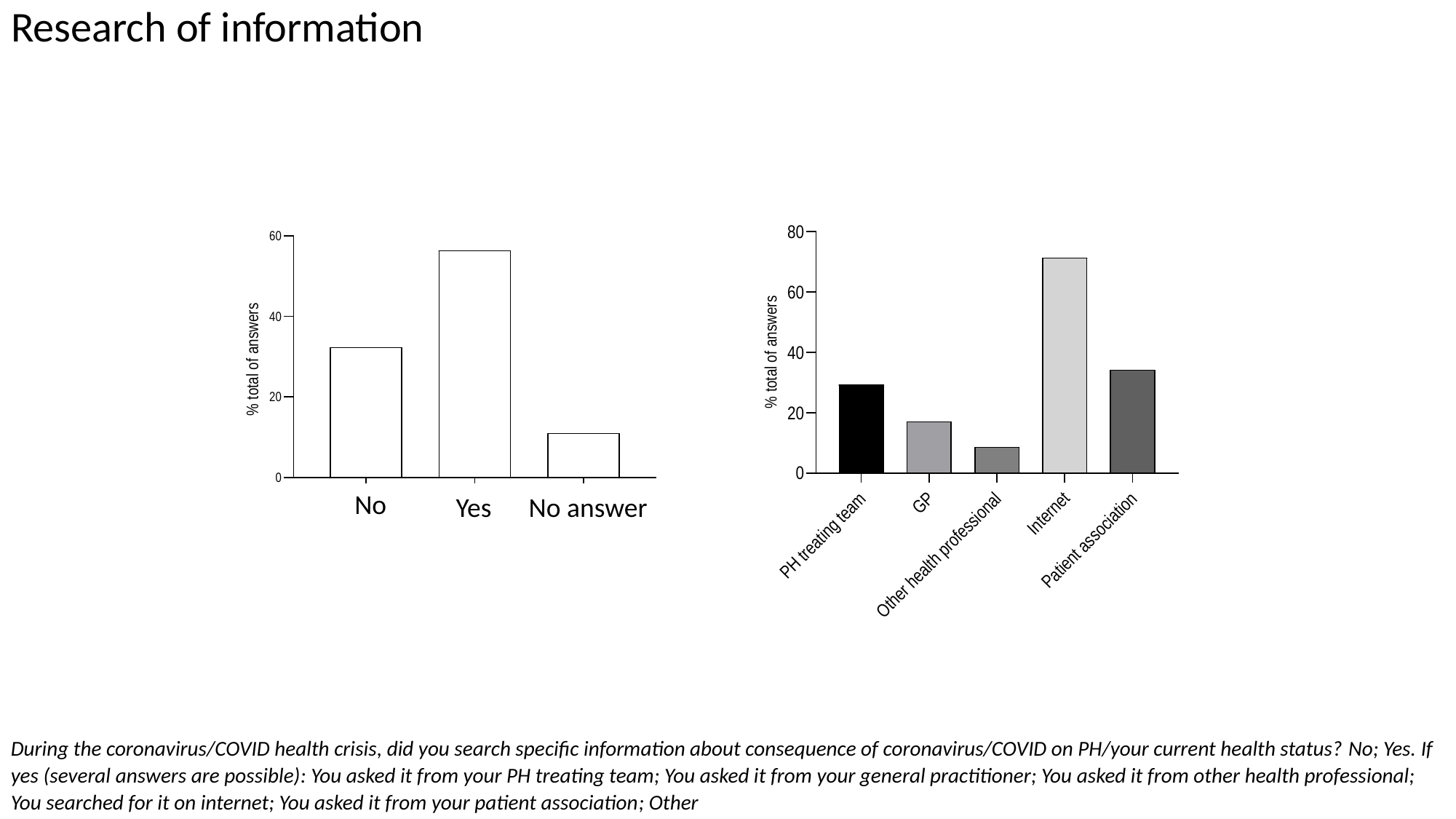

Research of information
No
 Yes
 No answer
During the coronavirus/COVID health crisis, did you search specific information about consequence of coronavirus/COVID on PH/your current health status? No; Yes. If yes (several answers are possible): You asked it from your PH treating team; You asked it from your general practitioner; You asked it from other health professional; You searched for it on internet; You asked it from your patient association; Other

## Slide 31
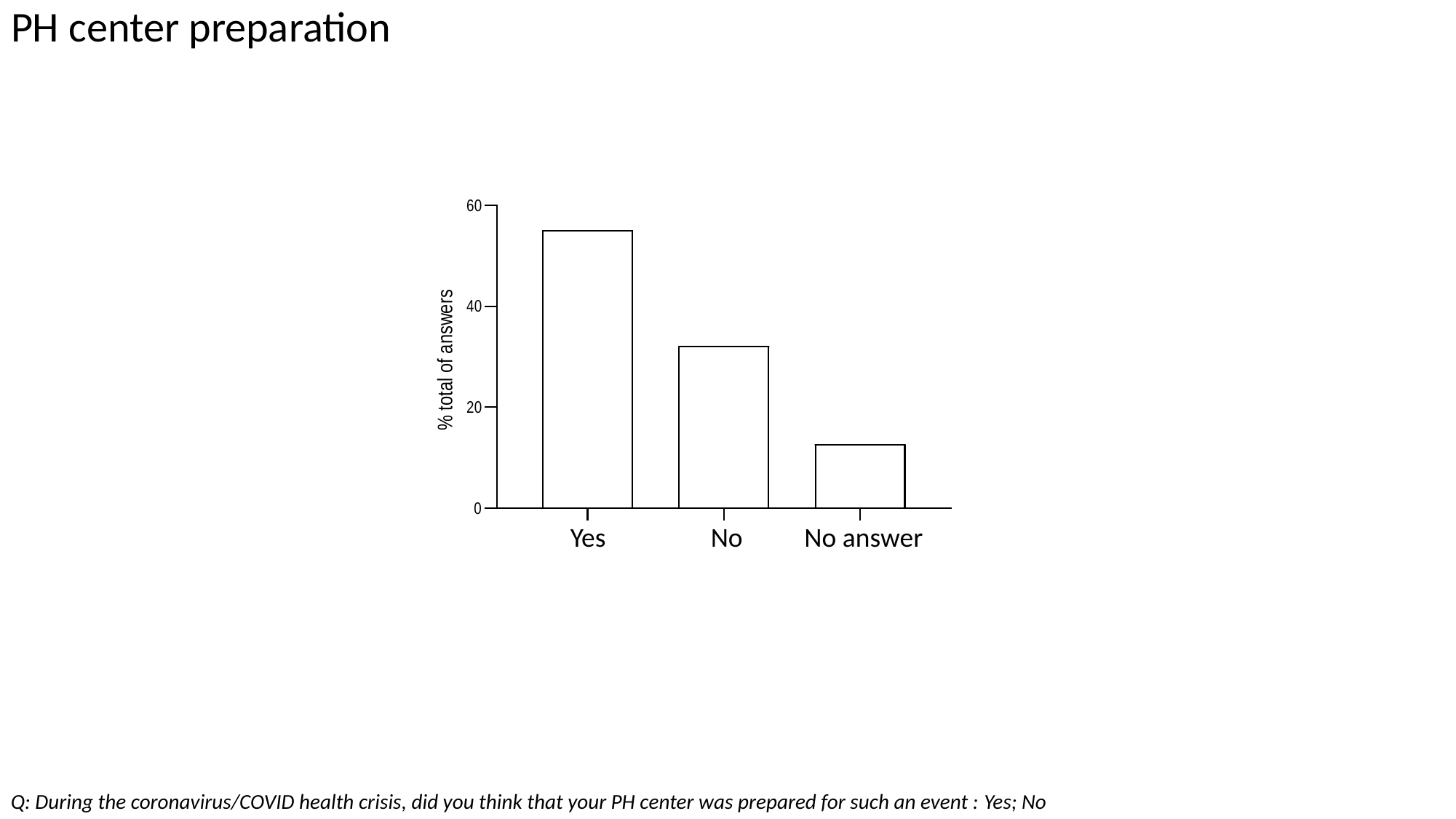

PH center preparation
Yes No No answer
Q: During the coronavirus/COVID health crisis, did you think that your PH center was prepared for such an event : Yes; No

## Slide 32
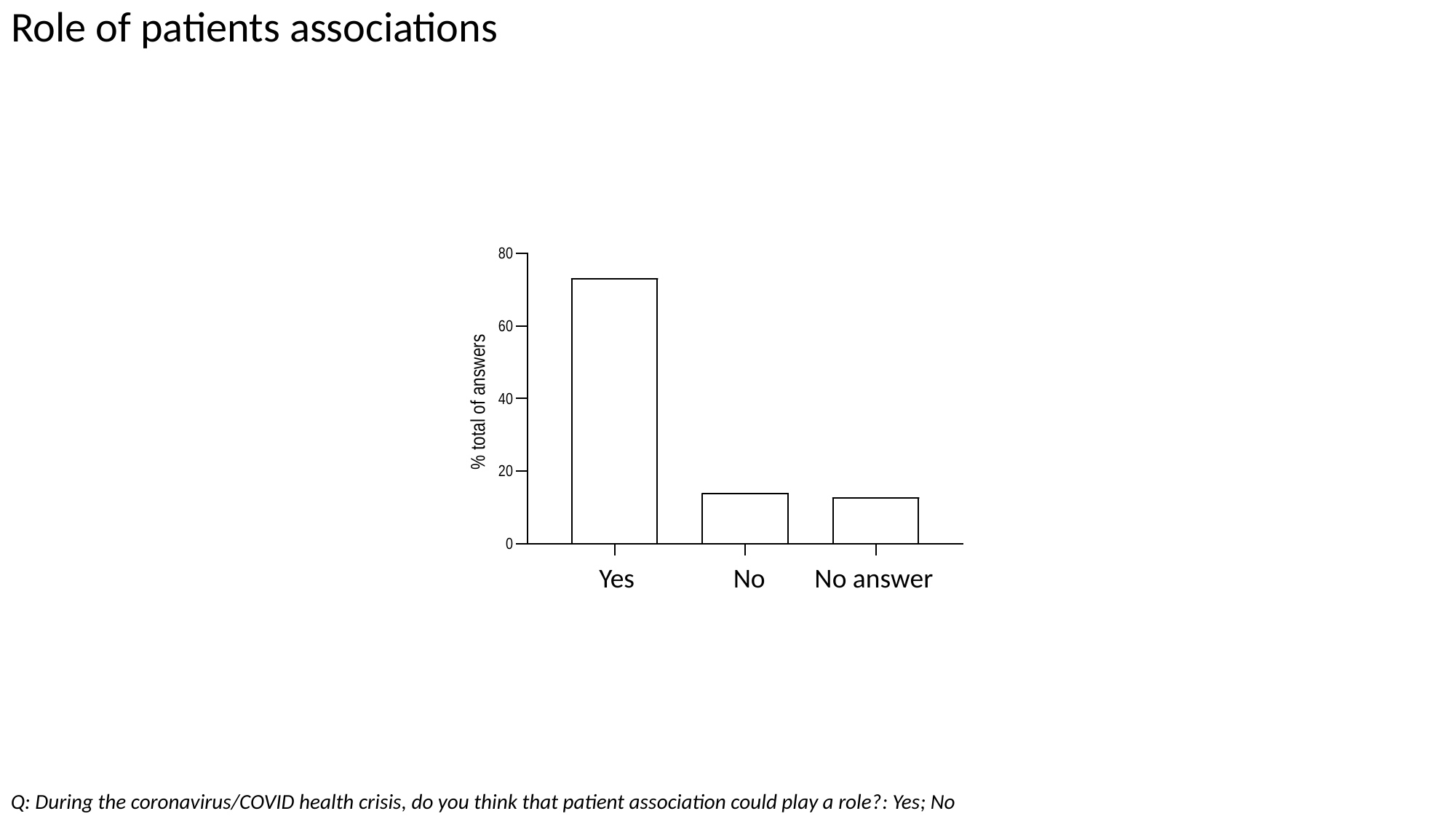

Role of patients associations
Yes No No answer
Q: During the coronavirus/COVID health crisis, do you think that patient association could play a role?: Yes; No
