# Supplementary material for: PH CARE COVID survey: an international patient survey on the care for pulmonary hypertension patients during the early phase of the COVID-19 pandemic
Source: Orphanet J Rare Dis. 2021 May 1;16:196. doi: 10.1186/s13023-021-01752-1 (PMC8087873; doi:10.1186/s13023-021-01752-1)
Supplement: Supplementary file 2 — Additional File 2. Complete results of the answers to the questionnaire. [file 13023_2021_1752_MOESM2_ESM.pdf]

PH patient care during the coronavirus health crisis survey (PHCARE-COVID survey)

https://phaeurope.app.box.com/s/jo299ygh5hmzt1jg7ty81juz06zpfaxi/folder/111050040897

Dear PH patients,

As a patient with PH, we are contacting you to hear about your medical care experiences during the COVID-19 pandemic. We would appreciate you answering this short questionnaire, so we can better understand how you may have been affected by the pandemic.

Most PH centers have been overloaded with extra work due to an increase in demand for care for COVID patients, which has made assuring regular care of PH patients significantly more difficult than usual. This has made it difficult for you to get in contact with your PH treating team/physicians, to obtain PH specific medications, and to come for outpatient visits and exams. Until now, specialists also do not know whether PH patients are more affected by coronavirus/COVID than the general population. They need a rapid and structured feedback from the members of the PH community concerning the cardiovascular and pulmonary consequences of coronavirus/COVID infection. This survey could contribute to organize health care for PH patients in the next few months. It could also provide information on how to improve health care in case of another dramatic and rapidly spreading event precluding optimal care of patients with chronic diseases.

*The **PHCARE-COVID survey** is run by ERN-Lung, a non-profit, registered virtual network involving healthcare providers across Europe. It aims to facilitate discussion on complex or rare respiratory diseases and conditions that require highly specialised treatment, and concentrated knowledge and resources. No commercial use of your details will be made at any time. The survey is a common initiative of **PHA Europe, ERN-Lung, ERS Assembly 13 on Pulmonary Vascular Diseases, ERS Clinical Research Collaboration PHAROS**, and **ESC WG on Pulmonary Circulation & Right Ventricular Function**. All of the information you share with us is completely confidential, it is used to create a collective analysis and your personal information will not be share with anyone.*

General information questionnaire

1. Are you answering this questionnaire as a…?
   - Patient
   - Parent of a child or adult with PH
   - Sibling of a child or adult with PH
   - Other family member
   - Other. Please specify

This PHCARE-COVID survey only accepts answers from people living with PH, their family or patient representative as participants.

2. Please select the country of your residence or of the residence of the patient you represent.

LIST OF COUNTRIES

3. Please indicate PH center where you or the patient you represent is followed up **(optional).**

LIST OF CENTERS + OTHER, please specify

4. How old are you or how old is the patient that you represent?
   - 0-10
   - 11-18
   - 19-29
   - 30-39
   - 40-49
   - 50-59
   - 60-69
   - 70-79
   - 80-89
   - ≥ 90

5. What is your gender, or the gender of the patient that you represent?
   1. Female
   2. Male
   3. Prefer not to say

6. What is the specific diagnosis of your PH disease or the PH disease of the patient you represent?
   a. Idiopathic pulmonary arterial hypertension
   b. Heritable/genetic pulmonary arterial hypertension
   c. Drug related pulmonary arterial hypertension
   d. Liver disease related pulmonary arterial hypertension
   e. Scleroderma or other inflammatory disease related pulmonary arterial hypertension
   f. Congenital heart malformation related pulmonary arterial hypertension
   g. Chronic thromboembolic PH
   h. I do not know/I am not sure
   i. Do not want to answer
   j. Other diagnosis (please specify)

*The **PHCARE-COVID survey** is run by ERN-Lung, a non-profit, registered virtual network involving healthcare providers across Europe. It aims to facilitate discussion on complex or rare respiratory diseases and conditions that require highly specialised treatment, and concentrated knowledge and resources. No commercial use of your details will be made at any time. The survey is a common initiative of **PHA Europe**, **ERN-Lung, ERS Assembly 13 on Pulmonary Vascular Diseases, ERS Clinical Research Collaboration PHAROS**, and **ESC WG on Pulmonary Circulation & Right Ventricular Function**. All of the information you share with us is completely confidential, it is used to create a collective analysis and your personal information will not be share with anyone.*

7. Which PH specific medication do you receive (several answers are possible)?
    a. Oral (tablets/pills, including Bosentan=Tracleer®/Ambrisentan=Volibris®/Macitentan=Opsumit®/Sildenafil=Revatio®/ Tadalafil=Adcirca®/Riociguat=Adempas®/Selexipag=Uptravi®):
    b. Intravenous (Epoprostenol=Flolan® and Veletri®):
    c. Subcutaneous (Treprostinil=Remodulin®):
    d. Inhaled (Iloprost=Ventavis® and Treprostinil=Tyvaso®):
    e. Trial medication, please specify
    f. Other. Please specify

8. Since how long are you followed up in a specialized center (please specify number of months)?
    TEXT BOX

<u>Coronavirus/COVID related questions</u>

1. During the coronavirus/COVID health crisis, have you experienced symptoms of coronavirus/COVID (several answers are possible)?
    a. Fever
    b. I have found it more difficult to breathe than normal
    c. I have felt more tired than usual
    d. My ankles have been more swollen than usual
    e. I have been coughing more than usual
    f. I had more chest pains than usual
    g. I have had skin problems (please specify):
    h. I have experienced problems with my ears, nose or throat
    i. I have lost some ability to smell or taste
    j. I have been coughing up of blood or blood-stained phlegm
    k. I have had arthritis (pain and/or swelling and/or redness of articulation)
    l. Cold
    m. Headache
    n. Muscle pain
    o. Sore throat
    p. Other (please specify) :

2. During the coronavirus/COVID health crisis, have you been tested for coronavirus?
    a. No
    b. Yes
        i. If yes the result is :
            1. Positive (you have coronavirus)
            2. Negative (you do not have coronavirus)
            3. The results were not conclusive or unknown

3. During the coronavirus/COVID health crisis, did you consult or have been in contact with health professionals about problems related to coronavirus/COVID?
    a. No
    b. Yes
        i. If yes, who have you been in contact with (several answers are possible) :
            1. General practitioner (GP)
            2. Treating physician for PH in your center
            3. Specialized nurse in your center
            4. Emergency department physician
            5. Intensive care unit department physician
            6. Lung specialist/cardiologist other than your PH physician
            7. Other. Please specify :

4. During the coronavirus/COVID health crisis, have you been hospitalised for coronavirus/COVID **suspicion** or coronavirus/COVID **disease**?
    a. No

b. Yes
    i. If yes, where were you hospitalised (several answers are possible)?
        1. In your PH center
            a. Emergency room
            b. General Ward
            c. Intensive care unit
            d. Other, specify
        2. In another institution/hospital
            a. Emergency room
            b. General Ward
            c. Intensive care unit
            d. Other, specify
        3. How long did you stay in the hospital (in days)?

PH related questions

1. During the coronavirus/COVID health crisis, did you have the feeling that your health status
   a. Improved
   b. Remained stable
   c. Deteriorated
      i. If it deteriorated (several answers are possible), do you think it was directly related to
         a. Coronavirus/COVID
         b. PH disease
         c. Lack of medication
         d. Lack of adequate follow up by a physician
         e. Anxiety/depression
         f. Lack of sleep
         g. Lack of activity
         h. Other. Please specify

2. During the coronavirus/COVID health crisis, did you consult or were in contact with health professionals about problems related to PH?
   a. Yes
   b. No
      i. If yes, who have you been in contact with (several answers are possible)?
         1. General practitioners (GP)
         2. Treating physician for PH in your center
         3. Specialized nurse in your center
         4. Emergency physician
         5. Intensive care unit physician
         6. Lung specialist/cardiologist other than your PH physician
         7. Other, specify
      ii. If no, why didn't you contact a health professional (several answers are possible)?
         1. Because it was not planned
         2. Because you were afraid to disturb physicians
         3. Because you were afraid to go to the hospital/general practitioner
         4. Because you thought that your problem was not important
         5. Other, specify

3. During the coronavirus/COVID health crisis, have you been hospitalized for problems related to PH?
   a. No
   b. Yes
      i. If yes, where were you hospitalized (several answers are possible)?
         1. In your PH center
            a. Emergency room
            b. General Ward

c. Intensive care unit
d. Other, specify
2. In another institution/hospital
   a. Emergency room
   b. General Ward
   c. Intensive care unit
   d. Other, specify
3. How long did you stay in the hospital (in days)?

4. During the coronavirus/COVID health crisis, did you have trouble with the following (several answers are possible)?
   a. Contacting your PH treating team
   b. Contacting health professionals other than your PH treating team
   c. Receiving adequate information related to the consequence of coronavirus/COVID on PH
   d. Receiving your PH specific medications
   e. Other. Please specify

5. During the coronavirus/COVID health crisis, did you experience cancellation of follow up appointments (several answers are possible)?
   a. PH treating physician consultation/appointment with your PH team
   b. Examination/tests related to PH (hospitalisation for check-up, echocardiography, right heart catheterisation, blood test…)
   c. No, I didn't experience any cancellation
      i. If you experienced cancellations, whose decision was it?
         1. It was the decision of my PH treating team
         2. It was my decision
         3. The decision was taken after discussion with my PH treating team
         4. Other, specify
      ii. Did you receive a new appointment?
         a. Yes
         b. No
            i. If you did not receive new appointments, did you receive other instructions (contact PH treating team at a specific moment, referral to other physician in your area, …):
            1. Yes
            2. No
            3. Other, specify

6. During the coronavirus/COVID health crisis, did you have (several answers are possible):
   a. Face-to-face contact with your PH team
   b. Video call (Facetime, WhatsApp, Skype, Zoom, other) with your PH treating team
   c. Phone call with your PH treating team
   d. Information received by email
   e. Information received by post

f. No contact at all although contact was planned or requested
g. No contact at all but there was no planned or requested contact

7. During the coronavirus/COVID health crisis, were your PH specific medications (several answers possible):
    a. Stopped because of shortage
        i. Yes
        ii. No
            1. If yes:
                a. How long was it stopped for?
                    i. Less than 1 week
                    ii. Between 1 week and 4 weeks
                    iii. More than one month
                b. Which type of medication was stopped?
                    i. Oral (=tablets/pills) medication
                    ii. Intravenous medication
                    iii. Subcutaneous medication
                    iv. Inhaled medication
                c. When you stopped receiving your medication, who did you inform?
                    i. Your PH treating team
                    ii. Your general practitioner
                    iii. Other health care practitioner
    b. Stopped by yourself
        i. Yes
        ii. No
            1. If yes, what was the reason (several answers possible)?
                a. Because of fear of aggravating risk of coronavirus/COVID
                b. Because you were not confident to pursue medication without optimal follow up
                c. Other, specify
    c. Modified but not interrupted (for example switch from a medication to another because your common medication was in shortage/not available)
        i. Yes
        ii. No
            1. If yes, please specify/describe how your medication was modified.
    d. Unchanged
    e. Stopped for other reasons, specify

<u>Previous vaccine related questions</u>

1. Have you received the PREVENAR 13 vaccine?
    a. No
    b. Yes
    c. Do not want to answer

2. Have you received the Bacillus Calmette-Guerin (BCG) vaccine?
    a. No
    b. Yes
    c. Do not want to answer

3. Have you received the seasonal flu vaccine?
    a. No
    b. Yes, this year
    c. Yes, last year
    d. Do not want to answer

Personal experience

The following questions are about your personal experience during the coronavirus pandemic.

1. In relation to your current PH health status, how do you feel (several answers are possible)?
    a. Anxious
    b. Upset (because of lack of care)
    c. Sad/depressed
    d. Abandoned
    e. Other, specify

2. Considering the medical care that you have received for PH, are you:
    a. Very satisfied
    b. Satisfied
    c. Neutral
    d. Dissatisfied
    e. Very dissatisfied
    f. Not applicable

3. Did you search for specific information about the consequences of coronavirus/COVID on your current health status?
    a. No
    b. Yes
        i. If yes, where did you search (several answers are possible)?
            1. Your PH treating team
            2. Your general practitioner
            3. Other health professional
            4. The internet
            5. Your patient association
            6. Other. Please specify

4. Do you think that your PH center was prepared for the coronavirus pandemic?
    a. Yes
    b. No
        i. If no, what are your recommendations/comments, specify (optional)

5. During the coronavirus/COVID health crisis, do you think that patient association could play a role?
    a. Yes
    b. No
        i. If yes, please specify how (optional)

6. Do you have any additional comments about your experience as a PH patient during the coronavirus/COVID health crisis? (optional)

    TEXT BOX

*The **PHCARE-COVID survey** is run by ERN-Lung, a non-profit, registered virtual network involving healthcare providers across Europe. It aims to facilitate discussion on complex or rare respiratory diseases and conditions that require highly specialised treatment, and concentrated knowledge and resources. No commercial use of your details will be made at any time. The survey is a common initiative of **PHA Europe**, **ERN-Lung, ERS Assembly 13 on Pulmonary Vascular Diseases, ERS Clinical Research Collaboration PHAROS**, and **ESC WG on Pulmonary Circulation & Right Ventricular Function**. All of the information you share with us is completely confidential, it is used to create a collective analysis and your personal information will not be share with anyone.*

Satisfaction questionnaire about PHCARE-COVID survey

1. Did the questions asked in this survey allow you to express your feelings about your patient experience or the experience of the patient you represent in the context of coronavirus/COVID healthcare crisis
    1. Very badly
    2. Quite badly
    3. Neutral
    4. Quite well
    5. Very well

2. Did you have remarks or recommendation about the **PHCARE-COVID** survey or other general comment to give about your experience of coronavirus/COVID health crisis as a PH patient, please specify? (optional)

3. How did you hear about the **PHCARE-COVID** survey (several answers possible)?
    1. At my health care centre/hospital, my doctor or a staff member told me about this survey
    2. Through a PH patient organization
    3. On the ERN-Lung website
    4. Through the ERN-Lung Member News
    5. On Twitter
    6. On Facebook
    7. On Linkedin
    8. On Instagram
    9. On another social network, specify
    10. Through a webinar
    11. At my health care centre/hospital through a poster, a leaflet or another on-site communication
    12. Through a friend or family member
    13. Other, specify

4. Do you want to receive the results of this survey by email?
    1. Yes
    2. No

5. What is your email address? (**optional**)

TEXT BOX

Please confirm your email address:

TEXT BOX

*The **PHCARE-COVID survey** is run by ERN-Lung, a non-profit, registered virtual network involving healthcare providers across Europe. It aims to facilitate discussion on complex or rare respiratory diseases and conditions that require highly specialised treatment, and concentrated knowledge and resources. No commercial use of your details will be made at any time. The survey is a common initiative of **PHA Europe, ERN-Lung, ERS Assembly 13 on Pulmonary Vascular Diseases, ERS Clinical Research Collaboration PHAROS**, and **ESC WG on Pulmonary Circulation & Right Ventricular Function**. All of the information you share with us is completely confidential, it is used to create a collective analysis and your personal information will not be share with anyone.*

6. Do you agree to be contacted by email to participate in potential other PH patient surveys? Note: You will be able to unsubscribe at any time from this contact list by sending a mail to phcarecovidsurvey@ern-lung.eu.
   i. Yes
   ii. No
